# Bioactive siRNA‐Based Liposomes Promoted Tendon‐Bone Healing in Osteoporotic Mice by Recovering the Stemness of CD248^+^ TSPCs

**DOI:** 10.1002/advs.202509883

**Published:** 2025-06-19

**Authors:** Yinhua Qian, Jinguo Zhu, Yanwei He, Haocheng Qin, Pingkang Qian, Bingfeng Sun, Haoqiang Huang, Chen Kuang, Quan Yang, Yuxi Ou, Rong Sun, Feng Xu, Xianwen Wang, Zhiwen Luo, Qing Wang

**Affiliations:** ^1^ Department of Orthopedics Kunshan Hospital of Chinese Medicine，Kunshan Hospital Affiliated to Nanjing University of Chinese Medicine No.388 Zuchongzhi South Road Kunshan Jiangsu 215300 China; ^2^ Department of Orthopedics Nantong Tongzhou Hospital of Traditional Chinese Medicine No.8 Jianshe Road Tongzhou Jiangsu 226300 China; ^3^ Department of Sports Medicine Huashan Hospital Fudan University Shanghai 200040 China; ^4^ Department of Rehabilitation Huashan Hospital Fudan University Shanghai 200040 China; ^5^ Department of Urology Huashan Hospital Fudan University Shanghai 200040 China; ^6^ Department of Radiation Oncology Jinling Hospital, Affiliated Hospital of Medical School Nanjing University Nanjing 210000 China; ^7^ School of Biomedical Engineering Research and Engineering Center of Biomedical Materials Anhui Provincial Institute of Translational Medicine Anhui Medical University Hefei 230032 China

**Keywords:** CD248, liposomes, osteoporosis, single cell, stemness, tendon‐bone healing, TSPCs

## Abstract

Osteoporosis significantly impairs tendon‐bone healing, increasing the risk of rotator cuff tears and postoperative retearing. Tendon stem/progenitor cells (TSPCs) are vital for tendon repair, with their stemness crucial to healing outcomes. This study investigated the role of CD248 in regulating TSPC stemness and assessed the therapeutic potential of si‐CD248‐loaded liposomes in promoting tendon‐bone healing under osteoporotic conditions. Single‐cell RNA sequencing (scRNA‐seq) identified increased TSPC populations, particularly a unique subcluster, TSPC‐0, with elevated CD248 expression in osteoporotic tendon samples. CD248^+^ TSPCs displayed reduced proliferation, increased apoptosis, and impaired migration, driven by altered FAK‐JAK‐STAT1 signaling. si‐CD248‐loaded liposomes were formulated and characterized, demonstrating efficacy in inhibiting CD248 expression, restoring TSPC stemness, and promoting tendon‐bone healing. In osteoporotic mice, liposome treatment significantly enhanced tissue regeneration, improving histological scores, collagen organization, and biomechanical properties. This study reveals that elevated CD248 expression negatively impacts TSPC stemness and impairs healing under osteoporotic conditions. Targeting CD248 using si‐CD248‐loaded liposomes effectively restores TSPC regenerative potential, representing a promising therapeutic strategy to enhance tendon‐bone healing in osteoporotic patients.

## Graphical abstract

In this study, it is found that osteoporosis leads to disturbances in the Extracellular Matrix (ECM)of tendons and thus causes a decrease in TSPC stemness, resulting in a rotator cuff that is easily damaged and prone to retearing after repair. Targeted intervention with si‐CD248‐loaded liposomes can restore TSPC stemness, promote tendon‐bone healing, and restore shoulder function.

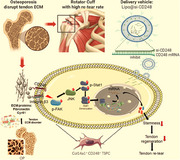

## 1 Introduction

In the geriatric population, rotator cuff tears (RCTs) are a predominant cause of shoulder discomfort^[^ ^1^, ^2^ ^]^ Open or arthroscopic cuff repair is an effective intervention for alleviating shoulder pain and improving joint functionality.^[^ ^3^, ^4^ ^]^ However, a high rate of retreatment after surgery remains a significant concern. Factors contributing to this risk include the size of the initial tear, tendon retraction, fatty infiltration, patient age, bone mineral density, and smoking habits.^[^ ^5^, ^6^ ^]^ Studies have identified osteoporosis as a risk factor not only for RCTs but also for postoperative retearing.^[^ ^7^, ^8^ ^]^ Reduced bone mineral density (BMD) and deteriorative osseous microstructure weaken suture anchor fixation, increasing risks of mechanical failure through screw pull‐out or loosening. Concurrently, dysregulated bone metabolism disrupts the osteotendinous healing microenvironment.

Therefore, managing osteoporosis in RCTs may help reduce the incidence of postsurgical retears. Osteoporosis is recognized as an independent risk factor for RCT.^[^ ^9^, ^10^ ^]^ Poor tendon‐bone integration is a primary reason for retearing and is influenced by factors such as bone density, anchor pull‐out strength, abnormal osteoclast activity, and the relationship between decreased estrogen levels and tendon‐bone healing.^[^ ^11^, ^12^, ^13^, ^14^ ^]^ However, previous clinical protocols have had limited effectiveness, and more treatment options are still needed.

Tendon stem/progenitor cells (TSPCs) play a critical role in tendon‐bone healing and repair due to their regenerative capabilities and ability to differentiate into various cell types required for tissue regeneration.^[^ ^15^ ^]^ TSPCs reside within tendon tissue and contribute to the maintenance and repair of tendon structure by producing essential extracellular matrix (ECM) components facilitating cellular turnover and also promote the formation of bone and cartilage at the tendon‐bone interface.^[^ ^16^, ^17^ ^]^ Their stemness, or their ability to self‐renew and differentiate, is pivotal for effective healing processes.^[^ ^16^, ^18^ ^]^ Studies have shown that maintaining TSPC stemness is crucial for tendon repair, as these cells orchestrate the regenerative processes necessary for integrating tendons with bone postinjury.^[^ ^17^, ^19^ ^]^ Furthermore, disruptions in TSPC function, often observed in conditions such as osteoporosis, lead to compromised tendon healing and increased susceptibility to retearing.^[^ ^17^, ^18^, ^20^ ^]^ The stemness of TSPCs—their capacity for self‐renewal and differentiation—is indispensable for tendon‐bone healing, particularly in rotator cuff repair, where these cells coordinate regenerative processes for functional tissue integration.^[^ ^16^, ^17^, ^18^ ^]^ However, inflammatory cascades triggered during rotator cuff injuries critically impair TSPC stemness. Proinflammatory cytokines (e.g., TNF‐α, IL‐1β) disrupt CD248+ TSPC self‐renewal, forcing pathological differentiation into fibrotic lineages instead of regenerative phenotypes.^[^ ^18^, ^20^ ^]^ This stemness loss creates a dysfunctional repair microenvironment, leading to defective tendon maturation and failed fibrocartilage zone reconstruction—hallmarks of poor postoperative outcomes.^[^ ^17^, ^20^ ^]^ In osteoporotic conditions, systemic bone loss amplifies local inflammation, synergistically depleting TSPC stemness and exacerbating retearing risks.^[^ ^17^ ^]^ Critically, impaired stemness perpetuates inflammation by reducing TSPC‐mediated resolution of inflammatory mediators, forming a self‐sustaining cycle of regeneration failure.^[^ ^19^ ^]^ Restoring TSPC stemness thus represents a therapeutic imperative to break this detrimental interplay in rotator cuff repair.

To enhance the regenerative potential of TSPCs and promote tendon‐bone healing, nanobiomaterials have emerged as promising tools due to their ability to modulate stem cell behavior at the molecular level.^[^ ^21^, ^22^, ^23^ ^]^ Among these, liposomes stand out for their unique advantages in biomedical applications. Liposomes, which are spherical vesicles composed of one or more phospholipid bilayers, closely mimic natural cell membranes, enabling them to efficiently interact with cellular structures and deliver therapeutic agents to specific target cells.^[^ ^24^, ^25^ ^]^ Their deformable bilayer structure proves particularly advantageous for navigating the complex fibrotic microenvironment characteristic of chronic rotator cuff injuries, enabling deep penetration into hypovascular tendon‐bone interfaces that conventional delivery systems often fail to reach. Their biocompatibility and ability to encapsulate both hydrophilic and hydrophobic molecules further enhance their utility in tissue engineering and regenerative medicine.^[^ ^26^, ^27^, ^28^ ^]^ In the context of tendon‐bone healing, liposomes offer several advantages. They can encapsulate siRNAs and other therapeutic agents, ensuring efficient delivery to target cells while protecting the siRNAs from enzymatic degradation. Additionally, liposomes facilitate the controlled and sustained release of therapeutic agents, maintaining effective concentrations at the injury site for prolonged periods.^[^ ^28^, ^29^, ^30^ ^]^ Their surfaces can also be functionalized to enhance cell‐specific targeting, thereby improving therapeutic efficacy and minimizing off‐target effects.^[^ ^31^ ^]^ These attributes collectively position liposomes as versatile and effective platforms for modulating stem cell properties and promoting tissue repair in challenging clinical scenarios.^[^ ^29^, ^32^, ^33^ ^]^

This study revealed a new tendon stem cell subcluster, CD248^+^ TSPC, with low stemness induced by osteoporosis, which contributes to a weakened rotator cuff that is susceptible to damage and prone to retearing after surgical repair. A novel targeted intervention using si‐CD248‐loaded liposomes, which effectively restored TSPC stemness, was developed. This approach promoted tendon‐bone healing and significantly improved shoulder function, demonstrating the potential of bioactive liposomes in enhancing the repair and regeneration of tendon tissues compromised by osteoporosis.

## 2 Results

### 2.1 Single‐Cell RNA Sequencing Analysis of Tendon Tissues from Osteoporotic (OP‐T) and Normal Control (NC‐T) Mice

**Figure 1:**
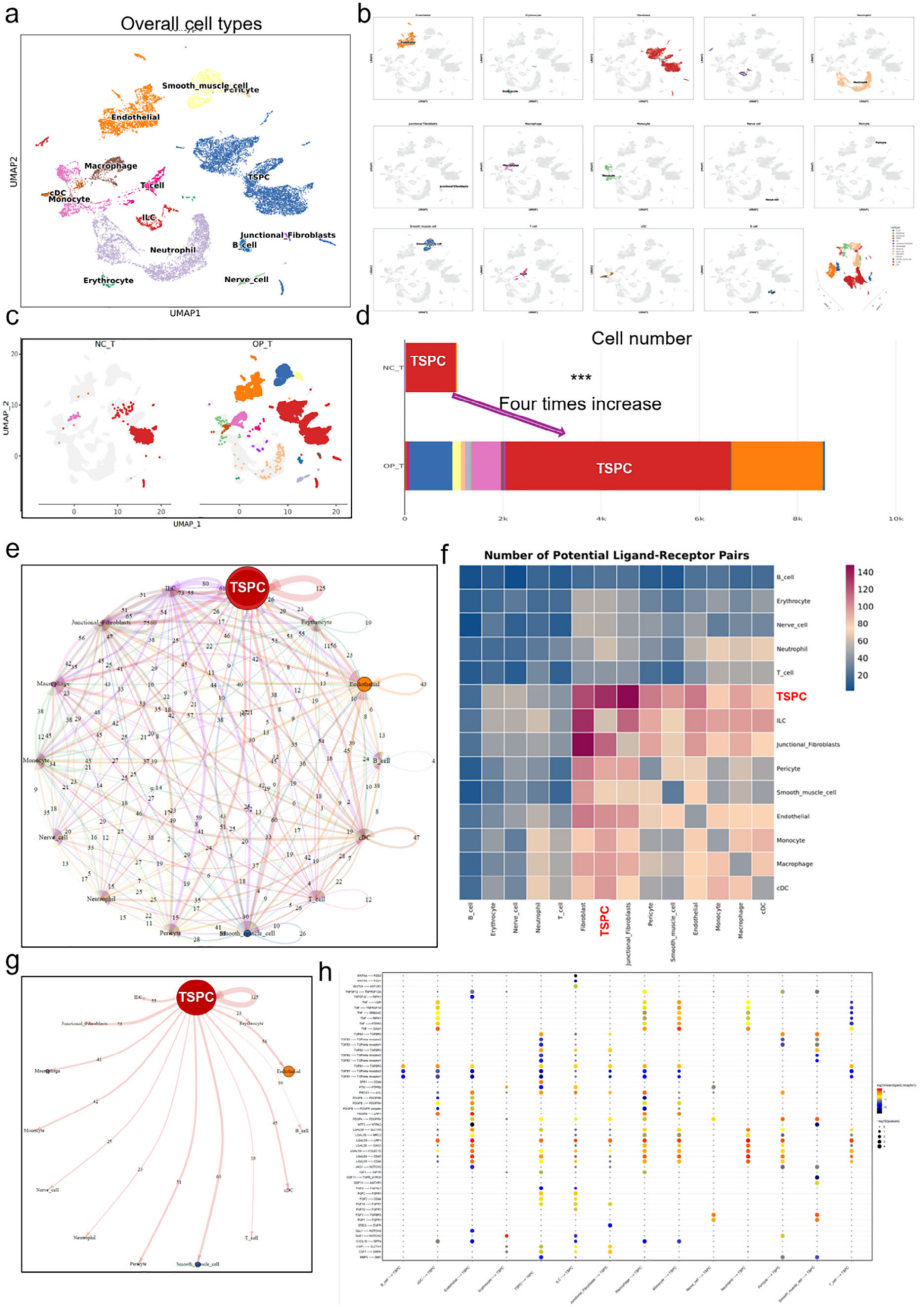
Single‐cell RNA sequencing analysis of overall cell types. a) UMAP plot showing cell types. This UMAP plot illustrates the clustering of different cell types identified through single‐cell RNA sequencing. Different colors represent various cell types, including tendon stem/progenitor cells (TSPCs), endothelial cells, macrophages, neutrophils, monocytes, fibroblasts, smooth muscle cells, nerve cells, and erythrocytes. b) Feature plots of subtypes in the UMAP plot. c) UMAP plot comparing cell distributions between the control and osteoporotic groups. This UMAP plot compares the distribution of cell types between the control group (NC_T) and the osteoporotic model group (OP_T), with a particular focus on the changes in TSPCs. d) Quantification of TSPC numbers. This bar chart quantifies the number of TSPCs in the control and osteoporotic model groups, showing a fourfold increase in TSPCs in the osteoporotic model group. e) Interaction network of TSPCs with other cell types. This network graph shows the potential ligand‒receptor interactions between TSPCs and other cell types. The direction of the arrows indicates the direction of signal transmission, with color and width representing the strength of interactions. f) Heatmap of potential ligand‒receptor pairs between cell types. This heatmap displays the number of potential ligand‒receptor pairs between different cell types, highlighting the interactions between TSPCs and other cell types. g) Detailed interaction map of TSPCs with other cell types. This interaction map provides a detailed view of the specific ligand–receptor pairs between TSPCs and other cell types, offering insights into cellular communication mechanisms. h) Dot plot of specific ligand–receptor pairs between cell types. This dot plot details the specific ligand–receptor pairs identified between TSPCs and other cell types, with dot size and color representing expression levels and significance.

To investigate the cellular composition and intercellular communication in tendon tissues under osteoporotic conditions, we performed single‐cell RNA sequencing on tendon samples from 5 osteoporotic mice (OP‐T group) and 5 normal control mice (NC‐T group) after three months of modeling. The quality control metrics for the single‐cell sequencing data, including the number of genes detected, total counts, and percentage of mitochondrial counts, are presented in Figure S1a (Supporting Information), ensuring the reliability of our dataset. A total of 10256 cells were collected and analyzed. Using marker‐based clustering, we categorized all cells into distinct populations. The UMAP plot in **Figure** **1**a displays the overall distribution of cell types, including TSPCs, endothelial cells, macrophages, smooth muscle cells, and other cell types. Figure S1b (Supporting Information) presents the correlation of various cell populations, and Figure S1c (Supporting Information) presents the cell cycle distribution and other parameters of various cell populations. Figure S1d,g (Supporting Information) displays the expression patterns of DEGs in TSPCs. Figure S1f (Supporting Information) presents the expression patterns of marker genes in various cell populations. A significant finding was the notable increase in the TSPC population in the OP‐T group compared to that in the NC‐T group. Figure 1d illustrates a fourfold increase in TSPC numbers in osteoporotic mice. This increase was visualized through comparative UMAP plots (Figure 1c), which revealed distinct clustering patterns between the two groups. To understand the potential interactions between TSPCs and other cell types, we conducted a ligand‒receptor pair analysis. Figure 1f presents a comprehensive network of potential interactions, highlighting the central role of TSPCs in the osteoporotic tendon microenvironment. Figure 1h provides a heatmap of the number of potential ligand‒receptor pairs, further emphasizing the enhanced communication pathways involving TSPCs in the OP‐T group. Figure 1e,g shows the linkage profiles between various cells, especially TSPCs. These results collectively demonstrate significant changes in the tendon cell population and intercellular communication under osteoporotic conditions, with a particular emphasis on the role of TSPCs. The increased TSPC population and altered communication networks suggest a potential mechanism through which osteoporosis may affect tendon biology and function.

### 2.2 Subcluster and Functional Analysis of TSPCs

We performed subcluster analysis on the TSPCs identified from the single‐cell RNA sequencing data. The UMAP plots in panel a show the expression patterns of selected genes across different TSPC subclusters, highlighting the diverse gene expression landscapes within these cells (Figure S2a, Supporting Information). Panel b depicts the proportional changes in TSPC subclusters between the NC‐T and OP‐T groups. Notably, there was a significant increase in the TSPC‐0 subcluster in the OP‐T group, suggesting a potential role of this subcluster in osteoporotic conditions (Figure S2b, Supporting Information). Panel c presents a heatmap of cell‐to‐cell correlations within TSPC subclusters, demonstrating high intracluster correlation and distinct intercluster differences. This confirms the distinct identities of the subclusters (Figure S2c, Supporting Information). Panel d shows a dot plot of pathway enrichment across TSPC subclusters, with the size of the dots representing the fraction of cells involved in each pathway and the color intensity indicating the mean expression level. Key pathways such as extracellular matrix organization and cell adhesion were prominently enriched in specific subclusters (Figure S2d, Supporting Information). The violin plots in panel e display the distribution of pathway activity scores for significant signaling pathways across different TSPC subclusters. These plots revealed distinct functional profiles among the subclusters (Figure S2e, Supporting Information). The heatmap in panel f shows DEGs across TSPC subclusters, with each row representing a gene and each column representing a subcluster. The color scale indicates relative gene expression levels, highlighting genes with significant differential expression patterns (Figure S2f, Supporting Information). Plots in panels e and g illustrate the expression patterns of subcluster‐specific marker genes, providing additional validation of the identified subclusters and illustrating the spatial distribution and intensity of these genes across the UMAP space (Figure S2e,g, Supporting Information).

**Figure 2:**
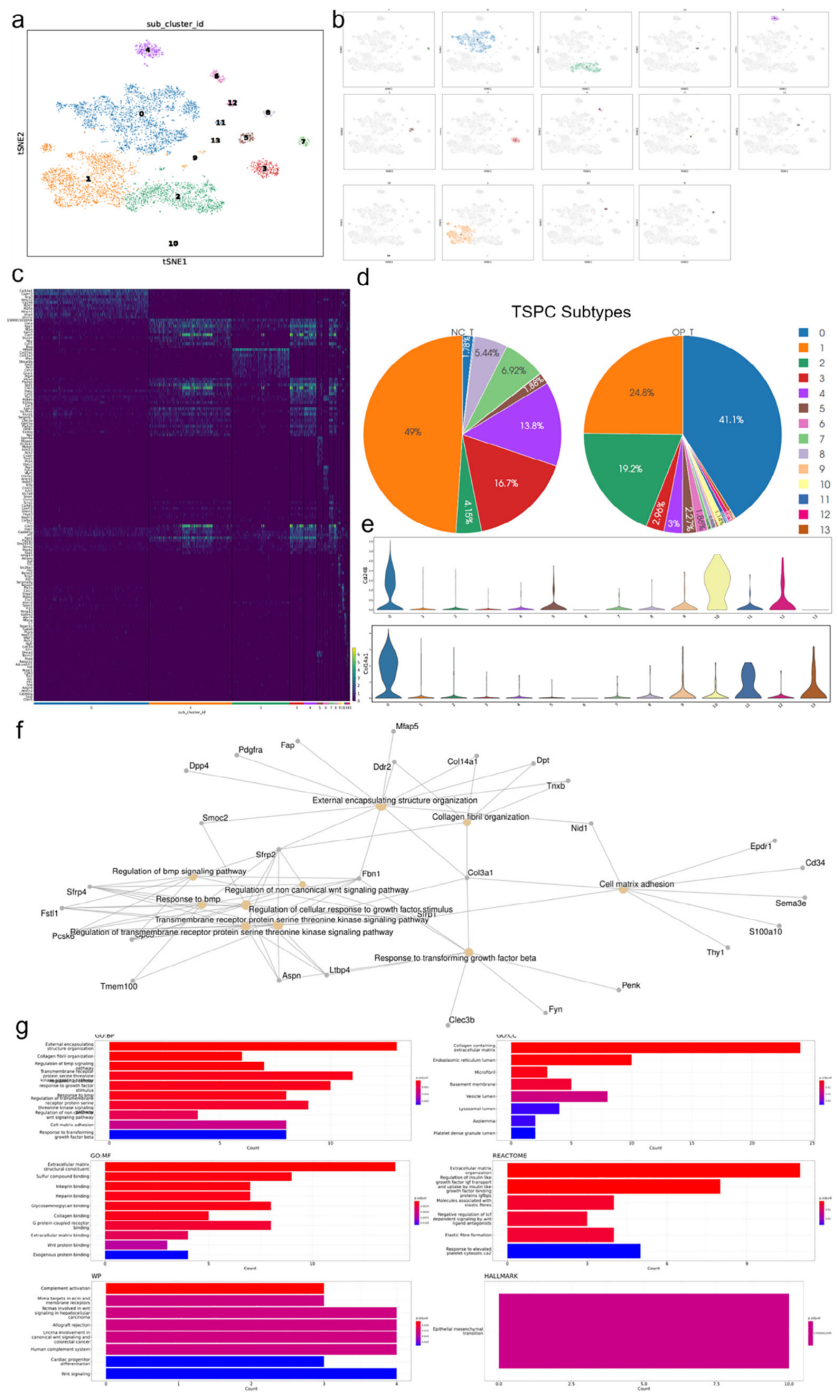
Subcluster analysis of single‐cell RNA sequencing data. a) t‐SNE plot showing subclusters of TSPCs. This t‐SNE plot illustrates the identification of distinct subclusters within the dataset, with each color representing a different subcluster (0–13). b) Feature plots of subclusters in t‐SNE. c) Heatmap of gene expression profiles across subclusters. The heatmap shows the gene expression profiles of the different subclusters, highlighting the distinct transcriptional signatures associated with each subcluster. d) Pie charts comparing the proportion of subclusters between the control and osteoporotic groups. These pie charts illustrate the relative abundance of each subcluster in the control group (NC_T) and the osteoporotic model group (OP_T), indicating significant shifts in cellular composition. e) Violin plots of gene expression in subclusters. The violin plots display the distribution of the top 10 gene expression levels for selected genes across the subclusters, providing insights into the functional heterogeneity within each subcluster. f) Gene Ontology (GO) and pathway enrichment network. This network diagram highlights the enriched GO terms and pathways associated with the DEGs in the subclusters, revealing key biological processes and signaling pathways. g) Bar charts of pathway enrichment analysis. These bar charts show the results of pathway enrichment analysis for the subclusters, including GO molecular function (GO‐MF), KEGG, Reactome, and hallmark pathways, underscoring the biological relevance of the identified subclusters.

The t‐SNE plot (**Figure** **2**a) revealed the presence of distinct subclusters within the TSPC population, each labeled with unique identifiers (e.g., 0, 1, 2). The feature plots (Figure 2b) show the expression of key marker genes across these subclusters, indicating heterogeneity within the TSPCs. Comparative analysis of the TSPC subclusters between the normal control (NC‐T) and osteoporotic (OP‐T) groups revealed significant differences in the distribution of these subclusters. The pie charts (Figure 2d) illustrate a notable shift in subcluster proportions, with certain subclusters, such as TSPC‐0, being markedly more abundant in the OP‐T group than in the NC‐T group. This suggests potential changes in TSPC functionality and state due to osteoporotic conditions. A heatmap of DEGs (Figure 2c) across the TSPC subclusters provides a detailed view of the gene expression landscape. Each row represents a gene, and each column represents a subcluster, with colors indicating the relative expression levels. This analysis highlights the specific gene expression patterns that distinguish each subcluster and suggests potential functional specializations. The violin plots (Figure 2e) display the expression levels of key marker genes across different TSPC subclusters. These plots emphasize the variability and specificity of gene expression within each subcluster, further validating the heterogeneity observed in the t‐SNE plots. To gain insight into the functional roles of the TSPC subclusters, we performed pathway enrichment analysis. The network graph (Figure 2f) illustrates the enriched biological pathways and gene ontologies associated with the DEGs in TSPC‐0. The key pathways included extracellular matrix organization, collagen fibril organization, and response to growth factors, indicating the involvement of TSPC‐0 in tissue remodeling and repair processes. Bar plots for Gene Ontology (GO), KEGG pathway, and other functional categories (Figure 2g) provide a quantitative view of the enrichment results for TSPC‐0. These plots show the top enriched categories, with significant representation of terms related to cell adhesion, signaling, and structural organization, reflecting the critical roles of TSPCs in maintaining tendon integrity and function under osteoporotic conditions.

Figure S3 (Supporting Information) provides a comprehensive functional and phenotypic analysis of the TSPC‐0 subcluster, highlighting its significant enrichment in extracellular matrix organization, cell signaling pathways, and specific protein domains. The analysis revealed that TSPC‐0 cells are actively involved in tissue remodeling and maintenance, with strong associations with clinical phenotypes such as Ehlers‒Danlos syndrome and potential links to cancer‐related genes. These findings underscore the critical roles of TSPC‐0 cells in the context of osteoporotic conditions and their broader biological significance. These results comprehensively detail the subcluster and functional analysis of TSPCs, highlighting their heterogeneity and the significant differences in subcluster composition and functionality between normal and osteoporotic conditions. These findings provide valuable insights into the role of TSPCs in tendon biology and their potential involvement in the pathophysiology of osteoporosis.

### 2.3 Pseudotime Analysis and Gene Expression Profiles of TSPC Subclusters

**Figure 3:**
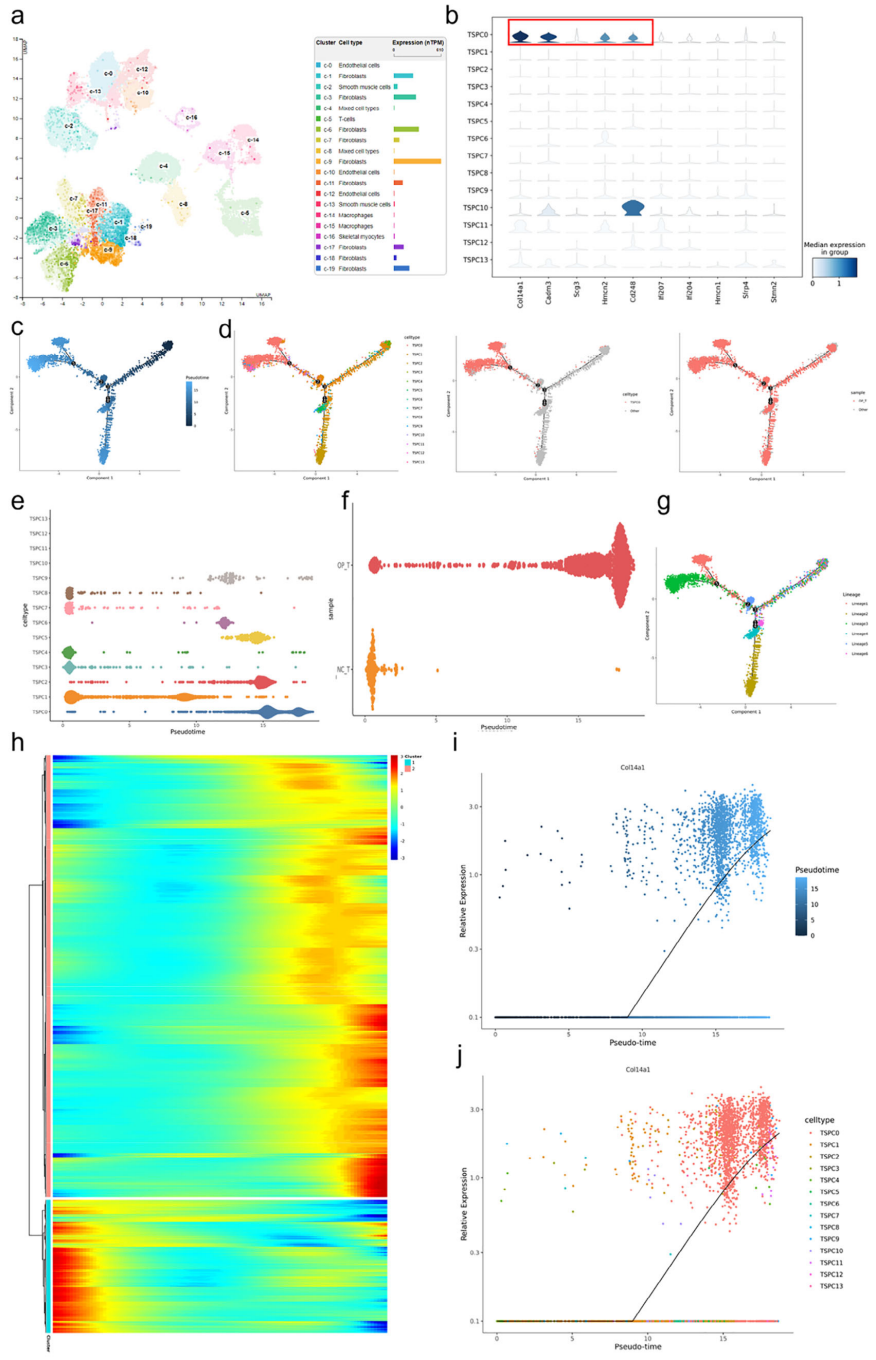
Pseudotime analysis of single‐cell RNA sequencing data. a) UMAP plot of cell clusters from the HPA database. This UMAP plot displays the clustering of cells into distinct groups based on their gene expression (CD248) profiles. Each color represents a different cell type, with annotations indicating specific clusters (e.g., C0, C1, C2). b) Violin plots of gene expression across TSPC subtypes. These violin plots illustrate the distribution of gene expression levels for the top 10 marker genes across different TSPC subtypes. The median expression in each group is highlighted to show variations in gene expression. c,d) Pseudotime trajectory analysis. e) Pseudotime trajectory analysis (colored by sample group). This trajectory plot is colored by sample group (e.g., NC_T and OP_T), showing how cells from different sample groups progress through pseudotime. f) Ridge plot of cell density along pseudotime. This ridge plot represents the density of cells along the pseudotime trajectory for each TSPC subtype, highlighting the distribution and transitions of subtypes over pseudotime. g) Pseudotime trajectory colored by lineage. This plot illustrates the pseudotime trajectory of cells colored by their inferred lineage, showing the developmental pathways of different cell lineages. h) Heatmap of gene expression along pseudotime. The heatmap displays the dynamic changes in gene expression along the pseudotime trajectory. Each row represents a gene, and each column represents a position along pseudotime, with colors indicating expression levels. i) Scatter plot of Col1a1 expression over pseudotime. This scatter plot shows the expression levels of the Col1a1 gene over pseudotime. Each dot represents a cell, colored by pseudotime, with a trend line indicating the overall expression trend. j) Scatter plot of Col1a1 expression by subcell type over pseudotime. Similar to panel i, this scatter plot displays Col1a1 gene expression over pseudotime, with cells colored according to their respective cell types, highlighting differences in expression patterns among cell types.

**Figure** **3**a shows the expression levels of CD248 across various cell subclusters using data from public single‐cell RNA sequencing databases. Notably, CD248 was predominantly expressed in fibroblast subclusters, highlighting its significance in these cell types (Figure 3a). Panel b shows the expression levels of the top 10 genes in the TSPC‐0 subcluster. The genes exhibited high expression levels in this subcluster, indicating their potential roles in defining the characteristics and functions of TSPC‐0 cells (Figure 3b). The pseudotime trajectory analyses (Figure 3c) depict the progression of TSPC subclusters over pseudotime, colored by various attributes such as cell type, sample group, and gene expression levels. These trajectories reveal the dynamic changes in gene expression as cells transition through different states, with TSPC‐0 emerging predominantly in osteoporotic (OP) conditions. Figure 3d shows the distribution of TSPC subclusters along pseudotime in both the NC‐T and OP‐T groups. The TSPC‐0 subcluster, which was significantly enriched in the OP‐T group, demonstrated a distinct pseudotime trajectory compared to the other subclusters. Figure 3e presents a heatmap illustrating the dynamic changes in gene expression over pseudotime. Each row represents a gene, and each column represents a pseudotime point, with colors indicating expression levels. The heatmap reveals temporal changes in gene expression associated with cellular transitions. Figure 3f shows scatter plots of Col14a1 expression over pseudotime, indicating significant upregulation of Col14a1 in the TSPC‐0 subcluster. The expression pattern of Col14a1 aligns with the pseudotime trajectory of TSPC‐0 cells, suggesting its involvement in osteoporotic conditions. Figure 3g shows the expression of CD248 across TSPC subclusters, with notable upregulation in the TSPC‐0 subcluster. This pattern is consistent with the gene expression dynamics observed in other osteoporotic‐related cell subclusters.

Figure S4 (Supporting Information) provides a comprehensive pseudotime analysis demonstrating the temporal gene expression dynamics within TSPC subclusters. The data illustrate how specific genes change in expression as cells progress through different states, with TSPC‐0 subcluster genes such as Col14a1 and CD248 showing distinct temporal expression patterns consistent with osteoporotic conditions. This analysis enhances our understanding of the molecular mechanisms underlying TSPC differentiation and function in the context of osteoporosis.

These results highlight the pseudotime analysis and gene expression profiles of TSPC subclusters, emphasizing the emergence of the TSPC‐0 subcluster under osteoporotic conditions. The consistent expression patterns of key genes such as Col14a1 and CD248 in TSPC‐0 and their alignment with the pseudotime trajectory underscore their potential roles in the pathophysiology of osteoporosis. This analysis provides valuable insights into the dynamic changes and functional implications of TSPC subclusters under osteoporotic conditions. In subsequent experiments, we named TSPC‐0 CD248^+^ TSPCs.

### 2.4 CD248^+^ TSPCs Showed Low Stemness In Vitro

**Figure 4:**
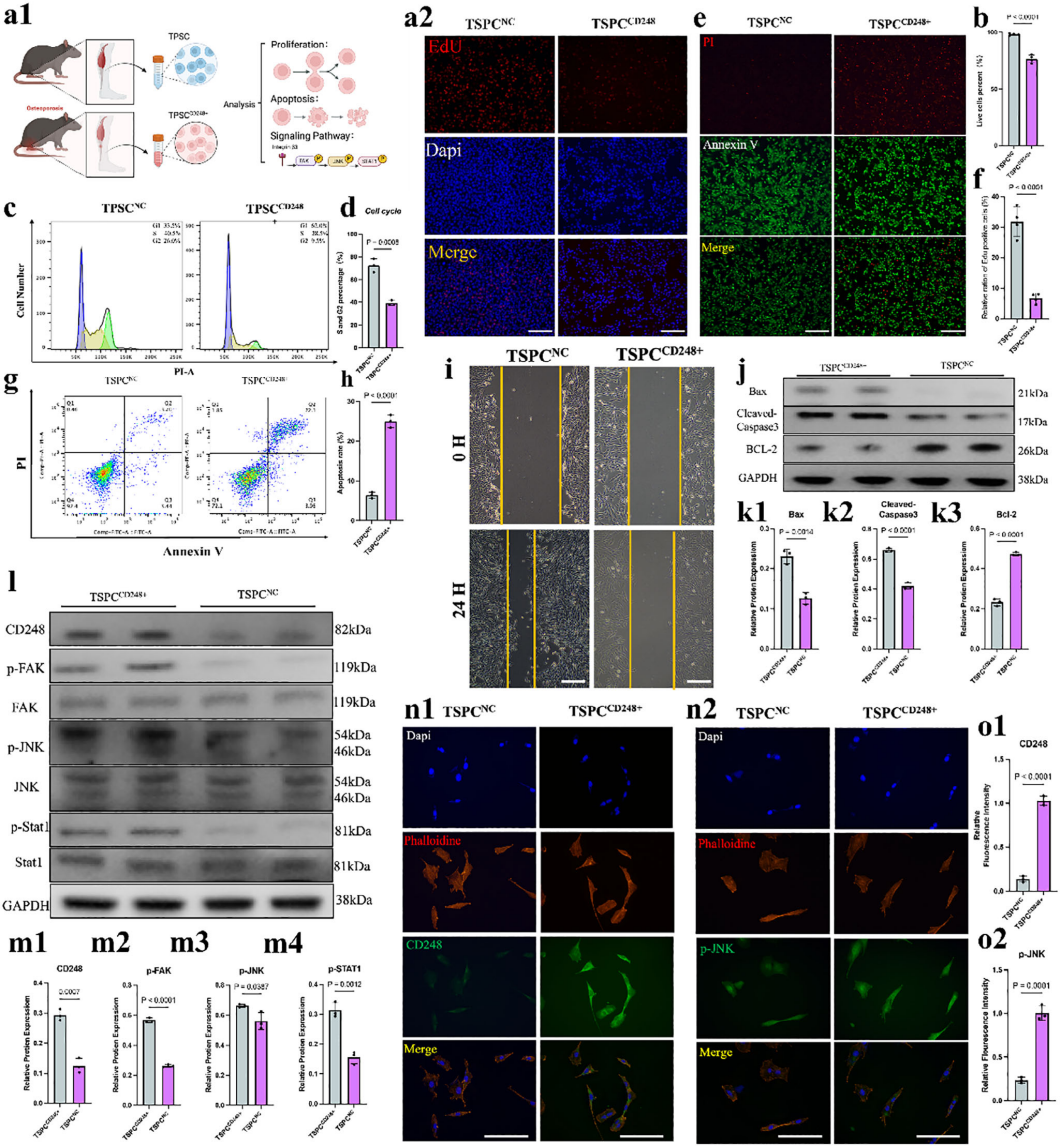
CD248+ TSPCs Showed Low Stemness In Vitro. a) a1‐Schematic diagram of cell processing. a2‐EdU staining for evaluating the proliferation rate of TSPCs and CD248‐positive TSPCs. The scale bars represent 100 µm. b) The proliferation rate of TSPCs was statistically analyzed (*n* = 3). The data are presented as the means ± SDs. c) Cell cycle analysis showing the proliferation status of TSPCs and CD248‐positive TSPCs. d) S and G2 phases of TSPCs and CD248‐positive TSPCs were statistically analyzed (*n* = 3). The data are presented as the means ± SDs. e) The percentages of apoptotic TSPCs and CD248‐positive TSPCs were assessed via live/dead staining. The scale bars represent 100 µm. f) The percentages of PI‐positive TSPCs and CD248‐positive TSPCs were statistically analyzed (*n* = 3). The data are presented as the means ± SDs. g) The percentages of apoptotic TSPCs and CD248‐positive TSPCs were assessed via apoptosis flow cytometry. h) The percentages of TSPC‐ and CD248‐positive TSPCs in the upper right and lower right quadrants were statistically analyzed (*n* = 3). The data are presented as the means ± SDs. i) Cell scratch analysis for evaluating the migration ability of TSPCs and CD248‐positive TSPCs. The scale bars represent 200 µm. j) Changes in apoptosis‐related protein expression in TSPCs and CD248‐positive TSPCs were analyzed. k) The relative protein expression of Bax, Bcl‐2, and cleaved caspase 3 was calculated after normalization to that of GAPDH (*n* = 3). The data are presented as the means ± SDs. l) The protein expression of CD248 and the protein phosphorylation of FAK, JNK, and STAT1 in TSPCs and CD248‐positive TSPCs were determined by Western blotting. m) The relative protein expression of CD248, p‐FAK, p‐JNK, and p‐STAT1 was calculated after normalization to that of GAPDH, FAK, JNK, and STAT1 (*n* = 3). The data are presented as the means ± SDs. n) Changes in the intensity of immunofluorescence staining for CD248 and p‐JNK (green) in TSPCs and CD248‐positive TSPCs. The scale bars represent 50 µm. o) The relative fluorescence intensities of CD248 and p‐JNK were calculated after normalization to those in the TSPCNC group (*n* = 3). The data are presented as the means ± SDs.

**Figure** **4**a,b shows the results of the EdU incorporation assays of CD248^+^ TSPCs. The images depict cells stained with EdU (red) and DAPI (blue), with the merged image indicating DNA synthesis activity. The low incorporation of EdU in CD248^+^ TSPCs suggested that the proliferative capacity of these cells was lower than that of control cells. Figure 4c,d presents the results of cell cycle analysis via flow cytometry. Histograms showing the distribution of CD248^+^ TSPCs across different phases of the cell cycle. The bar graph quantification reveals an increased proportion of cells in the G0/G1 phase and a decreased proportion in the S phase, further supporting reduced proliferative capacity. Figure 4e,f presents the Annexin V/PI staining results. Cells stained with Annexin V (green) and PI (red) are shown with merged images. An increase in Annexin V staining in CD248^+^ TSPCs indicates greater levels of early apoptosis than in control TSPCs. Figure 4g,h shows flow cytometry plots for apoptosis analysis using Annexin V/PI staining. The quantification bar graph indicates a greater percentage of apoptotic cells in the CD248^+^ TSPC population than in the control population. Figure 4j,k shows the western blot analysis of key apoptotic markers. The bands and corresponding quantification of Bax, BCL‐2, and Caspase3 indicate increased expression of apoptotic proteins in CD248^+^ TSPCs. Figure 4i shows the wound healing assay images taken at 0 and 24 h postcratch showing the migration capacity of CD248^+^ TSPCs. The bar graphs show significantly reduced wound closure in CD248^+^ TSPCs, indicating impaired migratory ability.

Figure 4l,m shows the western blot results for various signaling pathway proteins suggested by single‐cell analysis. The bands and quantification of proteins such as p‐JNK indicate altered signaling pathway activity in CD248^+^ TSPCs. Panels 4n and °present immunofluorescence staining for CD248 (green) and p‐JNK (green), along with DAPI (blue) and phalloidin (red). The merged images show colocalization of CD248 and p‐JNK, suggesting that CD248^+^ TSPCs have altered signaling pathways that may impact their stemness and functionality. In addition, we test potential downstream gene of JNK pathway—specific downstream target genes (such as Sox2, Oct4, Nanog) involved in the regulation of TSPC cell stemness. The results showed that Sox2, Oct4, Nanog gene expressions were significantly lower in the CD248^+^ TSPCs than in the normal group (Figure S5, Supporting Information).

Overall, Figure 4 demonstrates that CD248^+^ TSPCs exhibit reduced stemness characteristics in vitro. The decreased proliferative capacity, increased apoptosis, impaired migration, and changes in signaling pathways all indicate that compared with control cells, CD248^+^ TSPCs have compromised stem cell properties. These findings suggest that CD248 expression is associated with reduced stemness and may impact the regenerative potential of TSPCs in tendon repair and maintenance.

### 2.5 The Stemness of CD248^+^ TSPCs is Controlled by the CD248/FAK/JNK/Stat1 Signaling Axis

**Figure 5:**
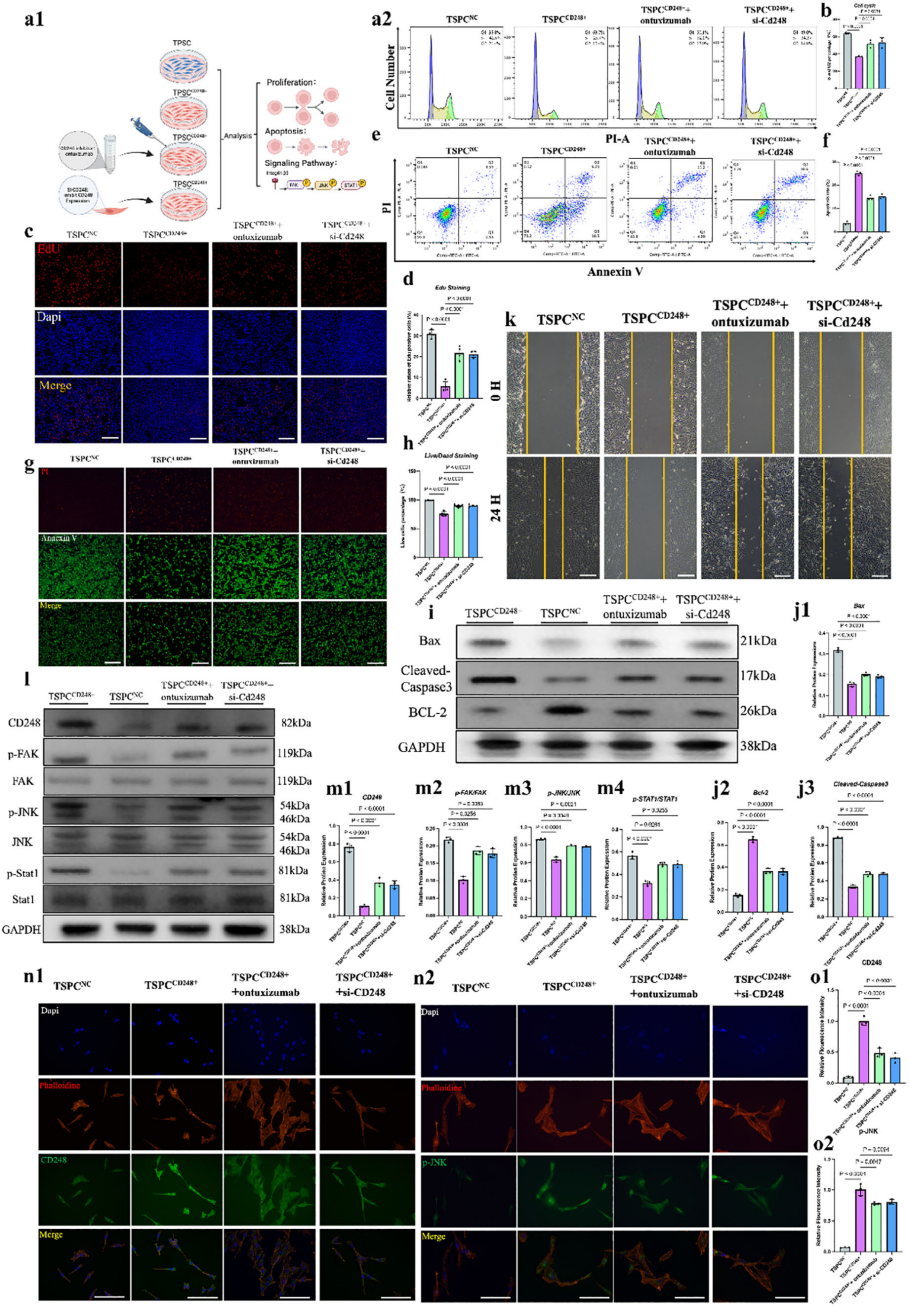
The Stemness of CD248+ TSPCs was Controlled by the CD248/FAK/JNK/Stat1 Signaling Axis. a) a1‐Schematic diagram of cell processing. a2‐Cell cycle analysis showing the proliferation status of TSPCs in the four different groups. b) The sum of the S and G2 phases of TSPCs in the four different groups was statistically analyzed (*n* = 3). The data are presented as the means ± SDs. c) EdU staining for evaluating the proliferation rate of TSPCs in the four groups. The scale bars represent 100 µm. d) The proliferation rate (red/blue) of TSPCs in the four groups was statistically analyzed (*n* = 3). The data are presented as the means ± SDs. e) The percentages of apoptotic cells among the four groups were assessed via apoptosis flow cytometry. f) The percentages of cells in the upper right (late apoptotic) and lower right (early apoptotic) quadrants among the four groups were statistically analyzed (*n* = 3). The data are presented as the means ± SDs. g) The percentages of apoptotic cells in the four groups were assessed via live/dead staining. The scale bars represent 100 µm. h) The percentage of PI‐positive cells (red) among the four groups was statistically analyzed (*n* = 3). The data are presented as the means ± SDs. i) Changes in the expression of apoptosis‐related proteins among the four groups were analyzed via Western blotting. j) Relative protein expression of Bax, Bcl‐2, and cleaved caspase 3 was calculated after normalization to that of GAPDH (*n* = 3). The data are presented as the means ± SDs. k) Cell scratch analysis for evaluating the migration ability of TSPCs in the four different groups. The scale bars represent 200 µm. l) The protein expression of CD248 and the protein phosphorylation of FAK, JNK, and STAT1 in the four groups were determined by Western blotting. m) The relative protein expression of CD248, p‐FAK, p‐JNK, and p‐STAT1 in the four groups was calculated after normalization to that of GAPDH, FAK, JNK, and STAT1 (*n* = 3). The data are presented as the means ± SDs. n) Changes in the intensity of CD248 and p‐JNK (green) immunofluorescence among the four groups are presented. The scale bars represent 50 µm. o) The relative fluorescence intensities of CD248 and p‐JNK were calculated after normalization to those in the TSPCNC group (*n* = 3). The data are presented as the means ± SDs.

Figure 4 shows that pathway changes in CD248^+^ TSPCs were consistent with the results of single‐cell transcriptome sequencing. Furthermore, we wanted to show that CD248, via its downstream pathway, indeed regulates the stemness of TSPCs (**Figure** **5**). Therefore, we used Ontuxizumab (a CD248 inhibitor) and si‐CD248 to inhibit CD248 expression in CD248^+^ TSPCs and then examined the downstream pathways and changes in stemness indicators. First, flow, value‐added, and scratch assays, as shown in Figure 5a–d,k, revealed that the value‐added and migratory abilities of CD248^+^ TSPCs were restored after inhibition of CD248. The apoptotic flow assay, WB assay, and apoptotic staining assay shown in Figure 5e,f,i,j demonstrated that the inhibition of CD248 in CD248^+^ TSPCs could be inhibited by a CD248 inhibitor after the inhibition of CD248, and the downstream pathway and stemness indices subsequently changed. Moreover, the apoptosis of CD248^+^ TSPCs was significantly reduced. Finally, WB and immunofluorescence staining experiments, as shown in Figure 5l–o, demonstrated that inhibition of CD248 expression resulted in a corresponding decrease in downstream pathway molecules, including phosphorylated FAK/JNK/Stat1 proteins; furthermore, significant downregulation of CD248, as well as p‐JNK, was also demonstrated via semiquantitative analysis. In addition, we tested stem markers such as Sox2, Oct4, Nanog. The results showed that Sox2, Oct4, Nanog gene expressions were significantly reversed by Ontuxizumab or si‐CD248 (Figure S7, Supporting Information).

Figure 5 demonstrates that the stemness of CD248^+^ TSPCs is regulated by the CD248/FAK/JNK/Stat1 signaling axis. The involvement of the CD248/FAK/JNK/Stat1 signaling pathway is evident through changes in the levels and localization of key signaling proteins. These findings suggest that targeting this signaling axis may be crucial for modulating the stemness and functional properties of CD248^+^ TSPCs.

### 2.6 Design, Characterization, and Biocompatibility of Lipo@si‐CD248

**Figure 6:**
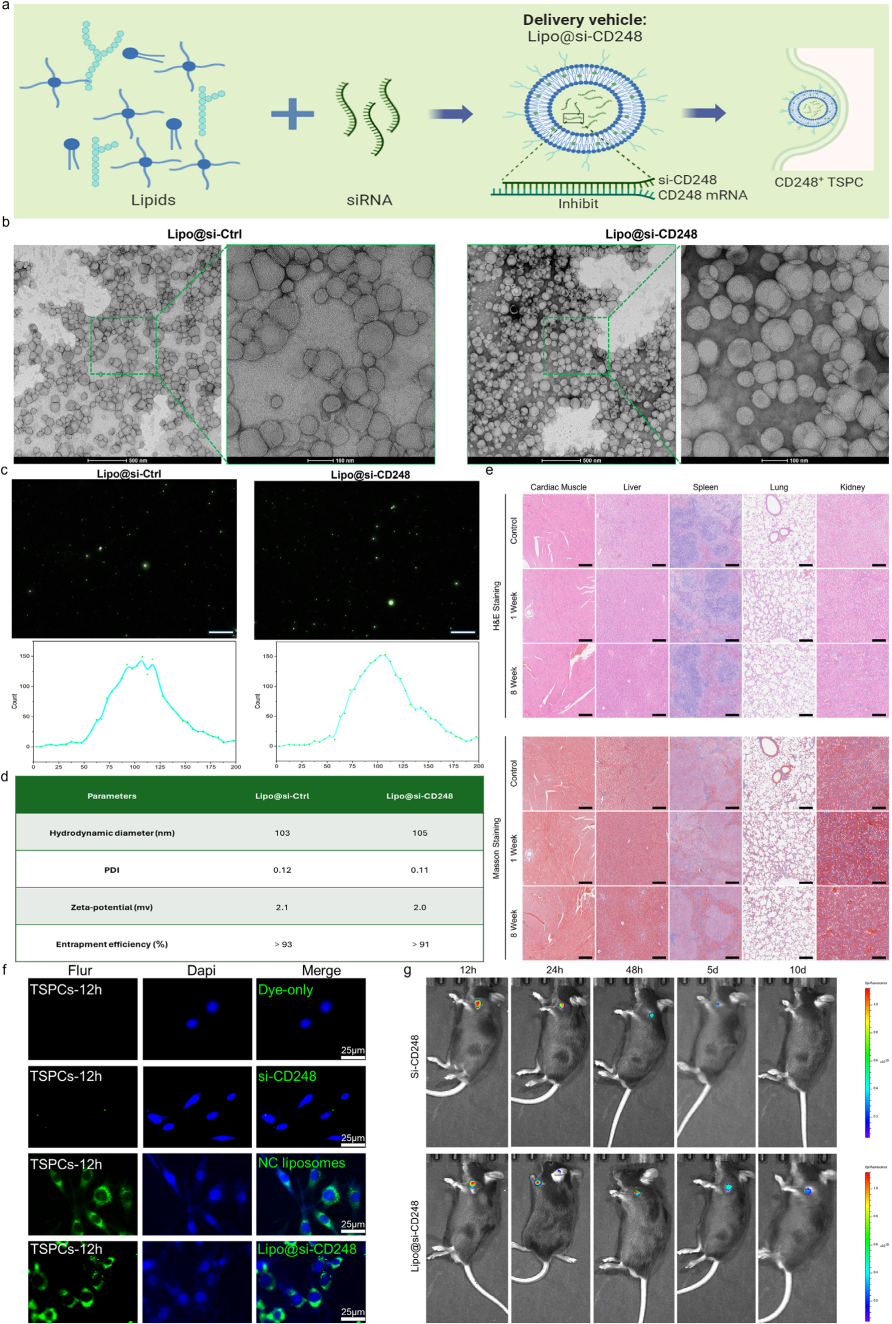
Characterization and efficacy of siRNA‐loaded liposomes for CD248+ TSPC delivery. a) Schematic of the Lipo‐siRNA Assembly and Delivery Mechanism. The schematic illustrates the assembly process of the siRNA‐loaded liposomes (Lipo@si‐CD248), where lipids are combined with siRNA to form the delivery vehicle. siRNA targets CD248 mRNA in CD248+ tendon stem/progenitor cells (TSPCs), inhibiting its expression. b) TEM images of liposomes. TEM images showing the morphology of Lipo@si‐Ctrl (left) and Lipo@si‐CD248 (right). Insets provide higher magnification images demonstrating the spherical shape and uniform size distribution of the liposomes. Scale bars represent 100 nm. c) NTA parameters and images of liposomes. Fluorescence microscopy images indicating successful loading of the siRNAs into the liposomes, as shown by the green fluorescence signals in both the Lipo@si‐Ctrl and Lipo@si‐CD248 samples. The scale bars represent 200 nm. d) Table of Liposome Characterization Parameters. The hydrodynamic diameter, PDI, zeta potential, and entrapment efficiency of Lipo@si‐Ctrl and Lipo@si‐CD248. Both liposome formulations show similar characteristics with high entrapment efficiency (>91%). e) Histological analysis of tissue sections treated with liposomes. Histological images showing tissue sections stained with H&E, indicating the biocompatibility and distribution of Lipo@si‐Ctrl and Lipo@si‐CD248 in different tissues. Scale bars represent 50 µm. f) The in vitro fluorescence imaging data of si‐CD248‐Flu and Lipo@si‐CD248‐Flu after incubation for 12h. The scale bars represent 25 µm. g) The in vivo fluorescence imaging data of si‐CD248‐Flu and Lipo@si‐CD248‐Flu after injection for 12h/24h/48h/5d/10d.

**Figure** **6** demonstrates the design, successful formulation, and thorough characterization of siRNA‐loaded liposomes (Lipo@si‐CD248). Figure 6a illustrates the process used to design and assemble Lipo@si‐CD248. Lipids combine with siRNAs to form liposomes, which then deliver siRNAs to inhibit CD248 mRNA expression in CD248^+^ tendon stem/progenitor cells (TSPCs). Figure 6b shows TEM images of the liposomes, revealing a uniform spherical morphology with an approximate diameter of 100 nm. The high‐resolution images confirmed the successful formation of liposomes. Figure 6c presents the NTA results, including images of the liposomes and size distribution profiles. NTA confirmed a hydrodynamic diameter of ≈100 nm for both Lipo@si‐Ctrl and Lipo@si‐CD248, demonstrating consistent particle size and distribution. Figure 6d summarizes the key parameters of the liposomes. The hydrodynamic diameter, polydispersity index (PDI), zeta potential, and entrapment efficiency are listed for both Lipo@si‐Ctrl and Lipo@si‐CD248. The liposomes exhibited a hydrodynamic diameter of ≈103–105 nm, low PDI values (0.11–0.12), and high entrapment efficiencies (>91%), indicating efficient siRNA encapsulation and stable formulation. Figure 6e displays histological images of tissue sections from animals treated with liposomes and stained with H&E and Masson's trichrome. The images show no significant tissue damage, inflammation or fibrosis, indicating good biocompatibility of the liposomes in vivo.

The results showed that si‐CD248 exhibited a significant sustained release effect in vitro after being encapsulated in liposomes, with siRNA sustained release within 72 h and a release rate of ≈80% in the first 24 h; However, siRNA without liposome protection is easily degraded rapidly((Figure S8, Supporting Information). In vitro fluorescence tracing experiments found that liposomes can effectively bind to TSPCs and be taken up by cells. After 12 h of incubation, significant binding and internalization phenomena can be observed, indicating good affinity between liposomes and cells (Figure 6f). In in vivo experiments, after intravenous injection of fluorescently labeled liposomes, the liposomes were significantly enriched in the target tendon bone interface area, and the fluorescence intensity reached its peak 12 h after injection, then gradually decreased, and remained in the target area for at least 10 days without significant diffusion to other tissues; In contrast, directly injected free siRNA did not show targeting in vivo and completely failed within 5 days, indicating that liposome drug delivery significantly improved the stability, targeting, and effective duration of siRNA (Figure 6g).

### 2.7 Lipo@si‐CD248 Enhanced the Stemness of CD248^+^ TSPCs by Inhibiting CD248 In Vitro

**Figure 7:**
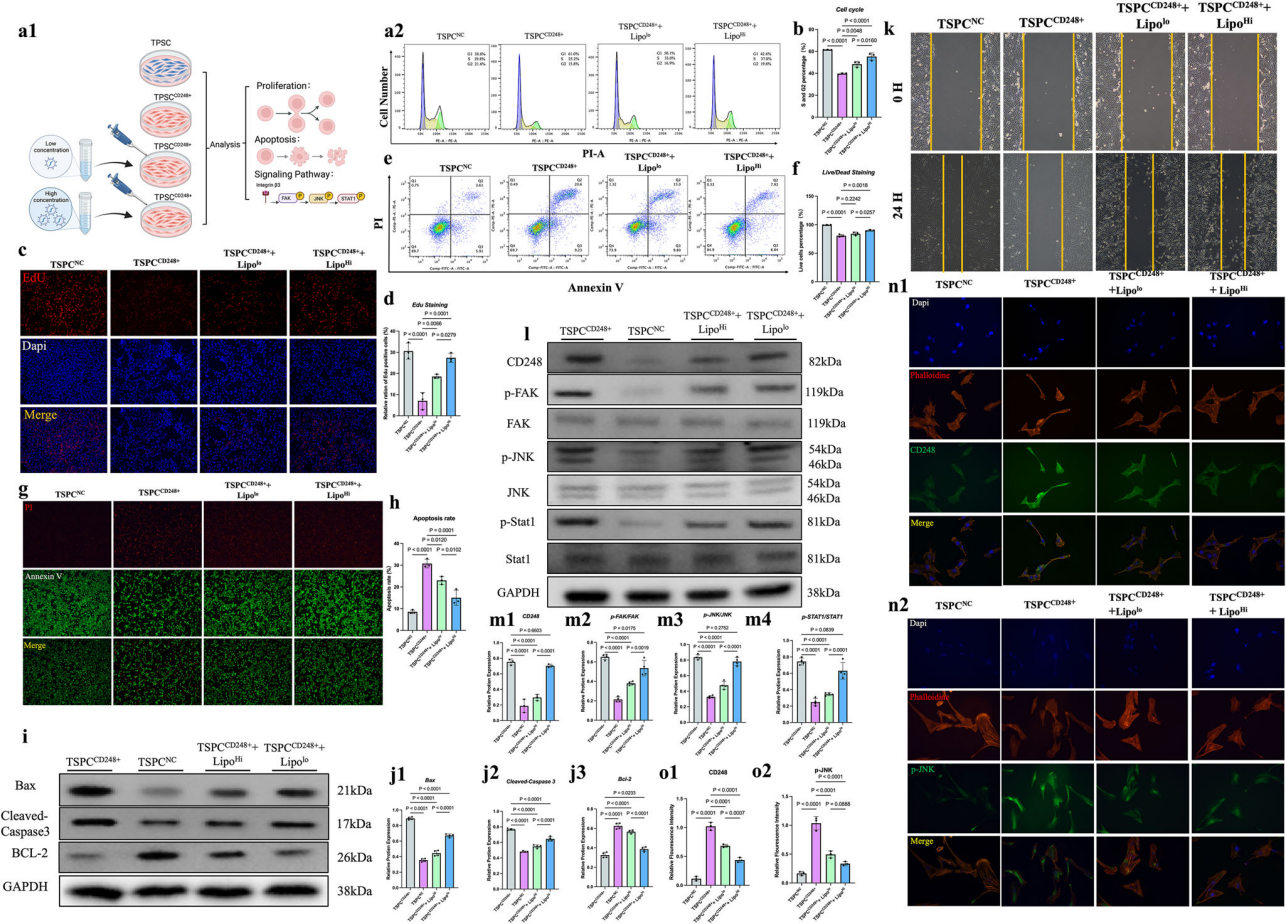
Lipo@si‐CD248 Enhanced the Stemness of CD248+ TSPCs by Inhibiting CD248 In Vitro a) a1‐Schematic diagram of cell processing. a2‐Cell cycle analysis showing the proliferation status of TSPCs in the four different groups. b) The sum of the S and G2 phases of TSPCs in the four different groups was statistically analyzed (*n* = 3). The data are presented as the means ± SDs. c) EdU staining for evaluating the proliferation rate of TSPCs in the four groups. The scale bars represent 100 µm. d) The proliferation rate (red/blue) of TSPCs in the four groups was statistically analyzed (*n* = 3). The data are presented as the means ± SDs. e) The percentages of apoptotic cells among the four groups were assessed via apoptosis flow cytometry. f) The percentages of cells in the upper right (late apoptotic) and lower right (early apoptotic) quadrants among the four groups were statistically analyzed (*n* = 3). The data are presented as the means ± SDs. g) The percentages of apoptotic cells in the four groups were assessed via live/dead staining. The scale bars represent 100 µm. h) The percentage of PI‐positive cells (red) among the four groups was statistically analyzed (*n* = 3). The data are presented as the means ± SDs. i) Changes in the expression of apoptosis‐related proteins among the four groups were analyzed via Western blotting. j) Relative protein expression of Bax, Bcl‐2, and cleaved caspase 3 was calculated after normalization to that of GAPDH (*n* = 3). The data are presented as the means ± SDs. k) Cell scratch analysis for evaluating the migration ability of TSPCs in the four different groups. The scale bars represent 200 µm. l) The protein expression of CD248 and the protein phosphorylation of FAK, JNK, and STAT1 in the four groups were determined by Western blotting. m) The relative protein expression of CD248, p‐FAK, p‐JNK, and p‐STAT1 in the four groups was calculated after normalization to that of GAPDH, FAK, JNK, and STAT1 (*n* = 3). The data are presented as the means ± SDs. *n*) Changes in the intensity of CD248 and p‐JNK (green) immunofluorescence among the four groups are presented. The scale bars represent 50 µm. o) The relative fluorescence intensities of CD248 and p‐JNK were calculated after normalization to those in the TSPCNC group (*n* = 3). The data are presented as the means ± SDs.

Lipo@si‐CD248 significantly reduced the protein expression of CD248 in TSPCs, as demonstrated by immunoblotting experiments (Figure S6, Supporting Information). The cell cycle distribution of the CD248^+^ TSPCs treated with Lipo@si‐CD248 compared to that of the untreated and control siRNA‐treated cells was analyzed via flow cytometry. The histograms indicate a decrease in the proportion of cells in the G0/G1 phase and an increase in the proportion of cells in the S phase among the Lipo@si‐CD248‐treated cells, suggesting enhanced proliferation (**Figure** **7**a,b). Figure 7c, d shows EdU incorporation assays with images of cells stained for EdU (red) and DAPI (blue). The merged images show increased EdU incorporation in CD248^+^ TSPCs treated with Lipo@si‐CD248, indicating enhanced DNA synthesis and proliferative activity. Quantification of the data revealed a lower percentage of apoptotic CD248^+^ TSPCs in the Lipo@si‐CD248 group than in the control group, indicating reduced apoptosis (Figure 7e,f). The images reveal decreased Annexin V (green) and PI (red) staining in CD248^+^ TSPCs treated with Lipo@si‐CD248, indicating decreased levels of early and late apoptosis. The bar graphs show the quantification of these observations (Figure 7g,h). Figure 7f shows the results of the wound healing assays. Images taken at 0 and 24 h postcratch showed enhanced migratory capacity in CD248^+^ TSPCs treated with Lipo@si‐CD248. The bar graphs show the quantified wound closure percentages, demonstrating improved migration.

Figure 7i,j,l,m presents the western blot analysis of apoptotic markers (e.g., Bax, BCL‐2, and Caspase3) and signaling proteins involved in the CD248/FAK/JNK/Stat1 axis. The blots and corresponding quantifications show increased expression of apoptotic markers in CD248^+^ TSPCs treated with Lipo@si‐CD248. In addition, this finding suggested that this signaling pathway is modulated by the treatment. Immunofluorescence staining of the actin cytoskeleton (phalloidin, red), nuclei (DAPI, blue), CD248 (green), and p‐JNK (green) is shown in panels 7n,o. The images show reduced colocalization of CD248 and p‐JNK in CD248+ TSPCs treated with Lipo@si‐CD248, indicating the involvement of the CD248/FAK/JNK/Stat1 axis in cell stemness signaling. These findings suggest that inhibiting CD248 with Lipo@si‐CD248 can significantly enhance the stem cell properties of CD248^+^ TSPCs, providing a potential therapeutic strategy for tissue regeneration.

### 2.8 Lipo@si‐CD248 Promotes Tendon‐Bone Healing After Rotator Cuff Injury in Osteoporotic Mice

**Figure 8:**
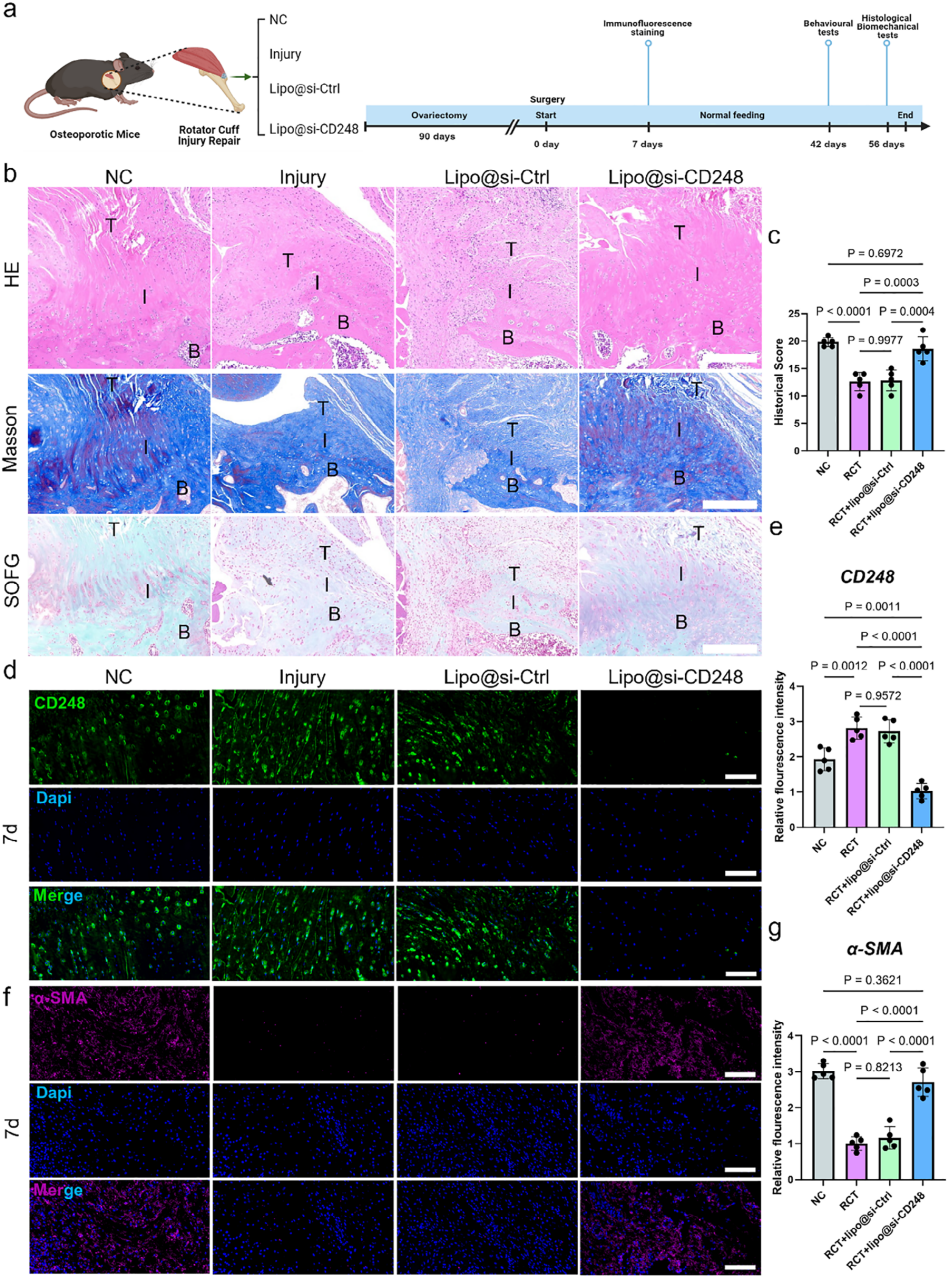
Lipo@si‐CD248 Promoted Tendon‐bone Healing after Rotator Cuff Injury in Osteoporotic Mice. a) Animal experiment flow chart. b) Histological staining results. The scale bars represent 200 µm. c) Quantification of histological staining. The data are presented as the mean ± SD. *p < 0.05, ***p < 0.001, ****p < 0.0001. d) Immunofluorescence staining results of CD248 on day 7. The scale bars represent 100 µm. e) Quantification of immunofluorescence staining. The data are presented as the mean ± SD. *p < 0.05, ***p < 0.001, ****p < 0.0001. f) Immunofluorescence staining results of α‐SMA on day 7. The scale bars represent 100 µm. g) Quantification of immunofluorescence staining. The data are presented as the mean ± SD. *p < 0.05, ***p < 0.001, ****p < 0.0001.

Subsequently, we conducted animal experiments. Mice were subjected to osteoporotic modeling for 3 months and then randomly assigned to four groups for experiments such as RCT modeling (**Figure** **8**a). After 3 months of osteoporotic modeling, microCT analysis was performed to assess the bone architecture and density in both normal control (NC) and ovariectomized (OVX) mice. Micro‐CT images revealed that the OVX mice exhibited markedly reduced bone density and deteriorated structural integrity compared to those of the NC mice (Figure S9a, Supporting Information). Quantitative analysis revealed significant decreases in the trabecular bone volume fraction (Tb.BV/TV), trabecular thickness (Tb.Th), and trabecular bone mineral density (Tb.BMD) in the OVX group. Additionally, there was a significant increase in trabecular separation (Tb.Sp) in the OVX group (Figure S9b, Supporting Information). These results confirmed the successful induction of osteoporosis in OVX mice, which was characterized by reduced bone mass and compromised bone structure (Figure S9a,b, Supporting Information). On the seventh day after RCT surgery, immunofluorescence staining was used to detect the expression levels of CD248. Compared to those in the other two control groups, the number of CD248‐positive cells in the Lipo@si‐CD248 group was significantly lower. This finding suggested that Lipo@si‐CD248 can inhibit the expression of CD248 in repair interface cells (including TSPCs) (Figure 8d,e).

At 8 weeks postsurgery, we further performed histological staining. HE, Masson, and Safranin O/Fast Green staining all showed better recovery in the Lipo@si‐CD248 group (Figure 8b,c). Specifically, for HE staining, at 8 weeks, the Lipo@si‐CD248 group displayed better‐organized collagen fibers accompanied by uniformly distributed cells along the collagen fibers (Figure 8c). Consequently, the histological score of the Lipo@si‐CD248 group was significantly greater than that of the Lipo@si‐Ctrl and Injury groups (Figure 8c). Compared with those in the Lipo@si‐Ctrl and injury groups, Masson's trichrome staining revealed a more orderly arrangement of collagen fibers and the formation of mature tendon‐to‐bone junctions in the Lipo@si‐CD248 group (Figure 8c). Moreover, for SOFG staining, the results showed a significantly larger metachromasia area at the interface in the Lipo@si‐CD248 group than in the other groups at 8 weeks (Figure 8c). Furthermore, since tenogenic cells play a significant role in matrix regeneration at the tendon‐bone interface, we evaluated the expression of their markers at the enthesis. Immunohistochemical staining at 1 week showed significantly stronger α‐SMA staining in the Lipo@si‐CD248 group than in the other groups (Figure 8f,g).

**Figure 9:**
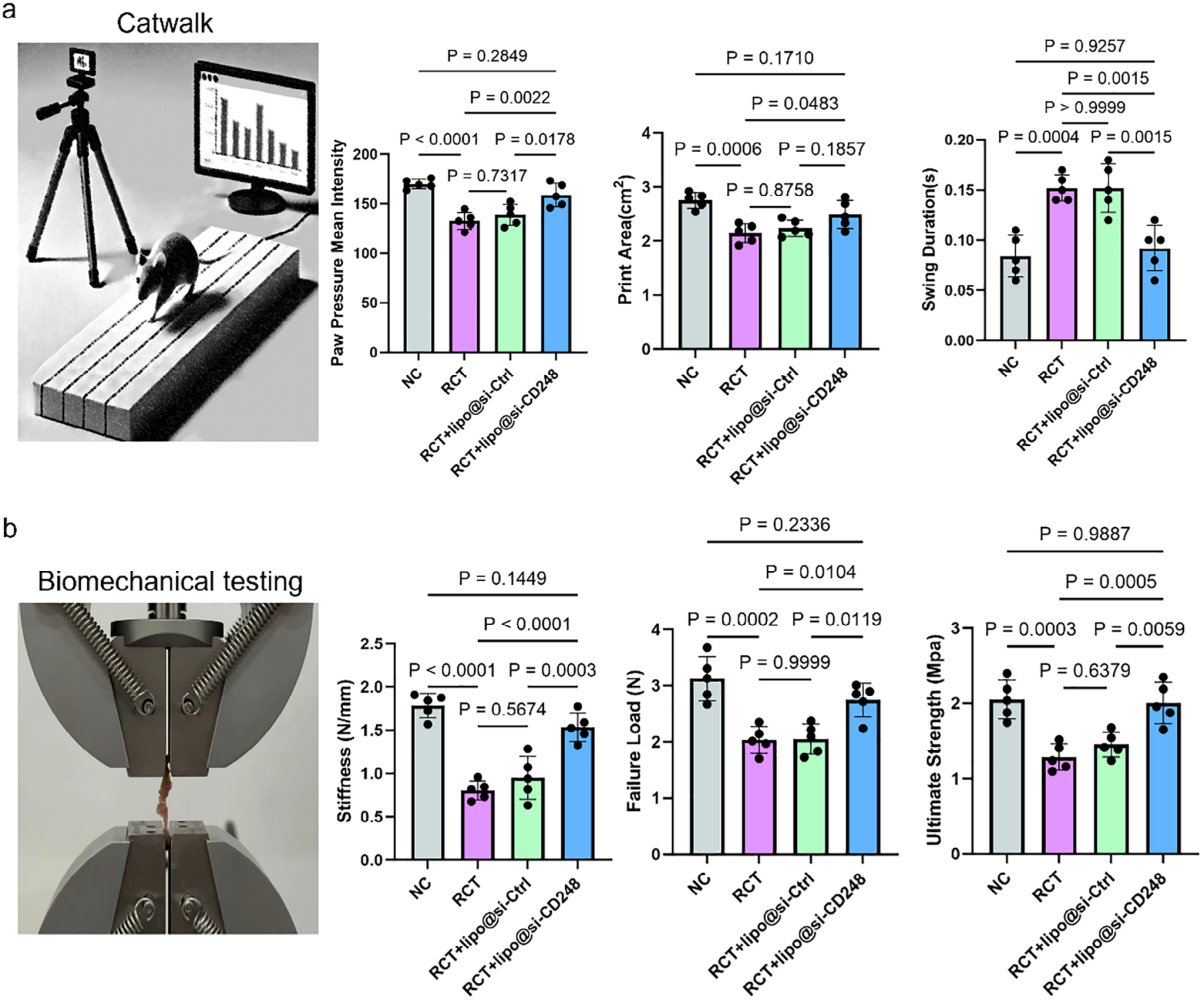
Lipo@si‐CD248 promoted shoulder functional recovery after rotator cuff injury in osteoporotic mice. a) Gait analysis results. b) Quantification of Gait. The data are presented as the mean ± SD. c) Results of biomechanical experiments. d) Quantification of biomechanical experiments. The data are presented as the mean ± SD.

Behavioral and biomechanical indicators of tissue repair can directly reflect the functional recovery of animals. Therefore, we conducted gait analysis at 6 weeks and biomechanical testing at 8 weeks. Mice use alternating support and the swing of their limbs to move forward. The function of the shoulder joint is crucial for the overall function of the forelimb, and abnormalities in shoulder joint behavior can be easily detected by gait analysis. Therefore, we used this method to evaluate the effect on tendon‐bone healing. At 6 weeks postrepair, the footprint area, average pressure intensity, and swinging time of the forelimbs in the Lipo@si‐CD248 group were greater than those in the Lipo@si‐Ctrl and Injury groups (**Figure** **9**a). Additionally, at 8 weeks, the maximum failure load of the Lipo@si‐CD248 group was significantly greater than that of the Lipo@si‐Ctrl and Injury groups (Figure 9b). Similarly, the stiffness and ultimate strength of the Lipo@si‐CD248 group were significantly greater than those of the Lipo@si‐Ctrl and Injury groups at 8 weeks (Figure 9b).

**Figure 10:**
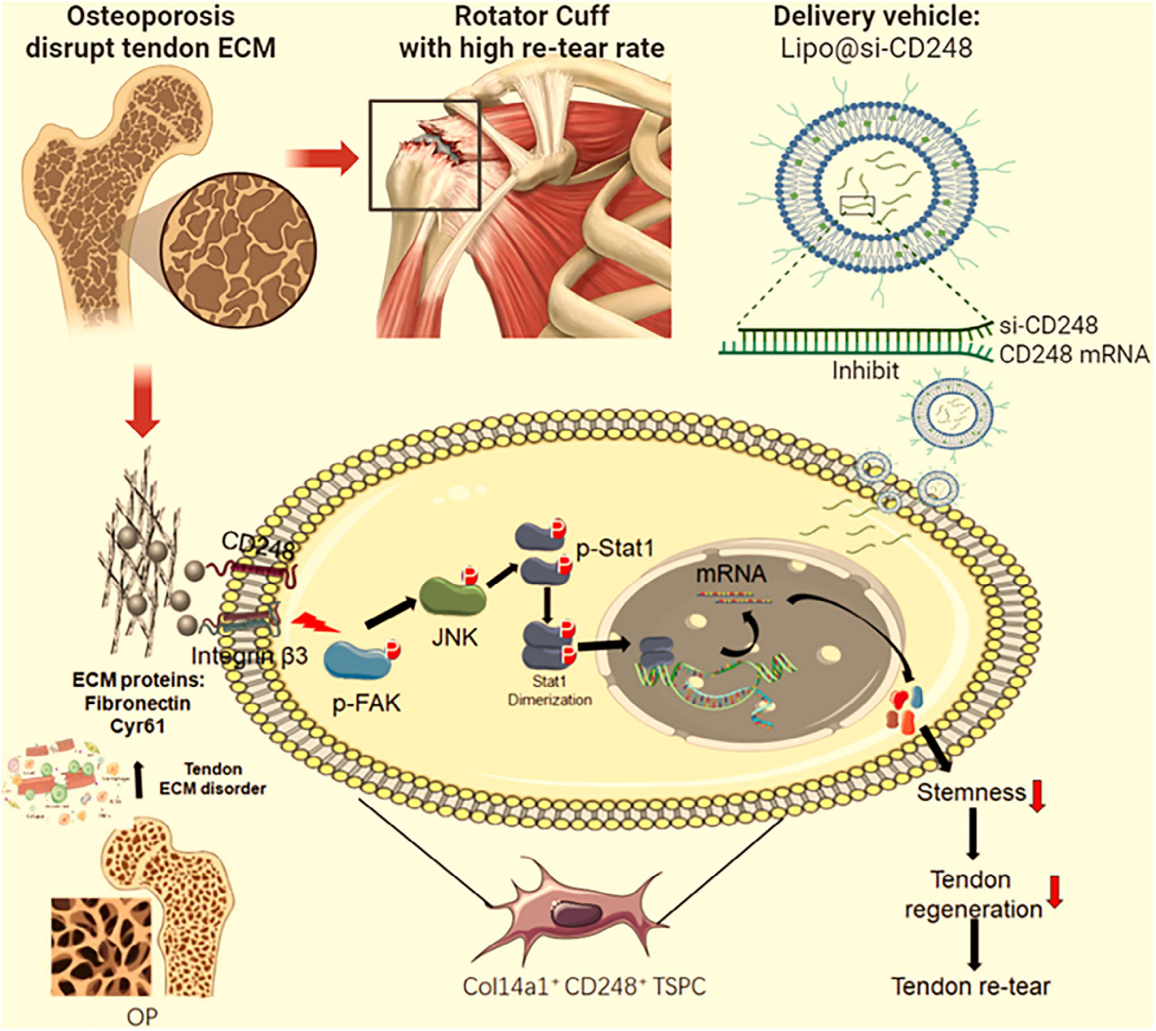
Schematic depiction of this work. In this study, we found that osteoporosis leads to disturbances in the ECM of tendons and thus causes a decrease in TSPC stemness, resulting in a rotator cuff that is easily damaged and prone to retearing after repair. Targeted intervention with si‐CD248‐loaded liposomes can restore TSPC stemness, promote tendon‐bone healing, and restore shoulder function.

In summary, these findings suggest that osteoporosis leads to disturbances in the ECM of tendons and thus causes a decrease in TSPC stemness, resulting in a rotator cuff that is easily damaged and prone to retearing after repair. Targeted intervention with si‐CD248‐loaded liposomes restored TSPC stemness, promoted tendon‐bone healing, and restored shoulder function (**Figure** **10**).

## 3 Discussion

Our study investigated the critical role of tendon stem/progenitor cells (TSPCs) and low‐stemness subtype CD248^+^ TSPCs in tendon‐bone healing, especially under osteoporotic conditions, and explored the therapeutic potential of si‐CD248‐loaded liposomes in enhancing TSPC stemness and promoting tendon repair. Our findings underscore the complex interplay between osteoporosis, TSPC functionality, and tendon healing and provide valuable insights into novel therapeutic strategies for improving surgical outcomes in osteoporotic patients.

Chuang et al. explored retear risk factors after rotator cuff repair surgery in a retrospective cohort study of 272 participants from 2004 to 2008. They reported a 22.8% postsurgical retear rate, with osteoporosis increasing the retear risk by 7.25 times and reducing bone mass by 4.38 times.^[^ ^7^ ^]^ Similarly, Chen Sheng et al.’s prospective study of 74 patients who underwent arthroscopic rotator cuff repair showed retear rates of 26.1% in the reduced bone mass group and 40.9% in the osteoporosis group, compared to 4.2% in those with normal bone mass.^[^ ^44^ ^]^ Hong et al. conducted a retrospective cohort study using Taiwan's insurance research database that included 21066 participants—3511 with osteoporosis and 17 555 without osteoporosis.^[^ ^8^ ^]^ Over a 7‐year follow‐up, they found that individuals with osteoporosis had a 1.79‐fold increased risk of developing rotator cuff tears (RCTs), with women being more susceptible than men. Stratified analyses confirmed that osteoporosis independently increased the risk of RCT, not merely serving as an age‐related surrogate, suggesting a link with abnormal bone metabolism.^[^ ^8^ ^]^

Poor healing at the tendon‐bone interface is a major cause of retearing after rotator cuff repair surgery.^[^ ^45^ ^]^ Osteoporosis affects tendon‐bone interface healing through several mechanisms: 1) Local osteoporosis of the humeral head impacts the immediate stability of rotator cuff repair. Bone density at the humeral head tubercle region is positively correlated with the pull‐out strength of the anchors. In cases of localized osteoporosis, anchor loosening is likely, compromising fixation strength and failing to provide an optimal microenvironment for tendon‐bone healing. Studies by Tingart et al.^[^ ^12^ ^]^ and Yakacki et al.^[^ ^46^ ^]^ demonstrated a direct relationship between bone density, the microarchitecture of the humeral head, and anchor pull‐out strength. Using a vitamin D deficiency‐induced osteoporotic mouse model, Angeline et al. reported that the osteoporotic group had a lower failure load and reduced bone and collagen fiber formation than the control group.^[^ ^47^ ^]^ 2) Excessive osteoclast activity hinders tendon‐bone healing. Rodeo et al. reported that abnormal osteoclast activity during the early healing phase (1–3 weeks postsurgery) impedes new bone formation and tendon‐bone healing.^[^ ^13^ ^]^ Xu et al., using an osteoporotic rabbit model, showed that early postoperative tendon‐bone interface healing deficits are linked to increased osteoclast activity.^[^ ^48^ ^]^ Schanda et al. also observed a high number of osteoclasts at the tendon‐bone interface, which correlated with reduced bone density and poorer biomechanical properties after rotator cuff repair.^[^ ^49^ ^]^ 3) A decrease in estrogen levels affects tendon‐bone healing. Osteoporosis often occurs with a decrease in sex hormones, particularly estrogen. Studies have shown that estrogen promotes tendon cell proliferation. Maman et al. reported that estrogen binds to estrogen receptors on tendon cells, enhancing their proliferation.^[^ ^14^ ^]^ Tanaka et al. used an ovariectomized rat model to study the effects of estrogen on tendon‐bone healing and reported that reduced estrogen levels led to compromised cartilage development and biomechanical properties, thus affecting tendon‐bone healing.^[^ ^50^ ^]^

Single‐cell RNA sequencing (scRNA‐seq) has become an indispensable tool for identifying pathological subpopulations within heterogeneous cell populations.^[^ ^51^, ^52^, ^53^, ^54^ ^]^ In our study, scRNA‐seq allowed us to dissect the cellular composition of tendon tissues from osteoporotic (OP‐T) and normal control (NC‐T) mice, revealing significant shifts in cell populations under osteoporotic conditions. The detailed analysis highlighted a notable increase in tendon stem/progenitor cells (TSPCs) in the OP‐T group, particularly the emergence of a distinct subcluster, TSPC‐0, which exhibited elevated expression of CD248. High expression of CD248 in TSPC‐0 cells was further analyzed to understand its impact on cell stemness.CD248, also known as endosialin, is associated with various pathological conditions and is a marker for certain fibroblasts. Its overexpression drives pathological inflammation by enhancing extracellular matrix (ECM) remodeling and activating pro‐inflammatory pathways, including NF‐κB and MAPK signaling, which elevate cytokines like IL‐6 and TNF‐α. Additionally, CD248 suppresses stemness‐related transcription factors (e.g., Oct4, Sox2) while promoting fibrotic differentiation via TGF‐β signaling, ultimately impairing the regenerative potential of stem/progenitor cells in degenerative tissues. These mechanisms link CD248 to chronic inflammation and reduced cellular plasticity in disease microenvironments. Our in vitro experiments demonstrated that CD248+ TSPCs exhibited reduced stemness, characterized by decreased proliferative capacity, increased apoptosis, and impaired migratory ability. Flow cytometry and EdU incorporation assays indicated decreased DNA synthesis, while Annexin V/PI staining and western blot analyses revealed increased levels of apoptotic markers in CD248+ TSPCs compared with those in controls. Moreover, pseudotime trajectory analysis provided insights into the dynamic changes in gene expression associated with CD248+ TSPCs under osteoporotic conditions. The expression patterns of key genes such as Col14a1 aligned with the pseudotime trajectory of TSPC‐0 cells, suggesting their involvement in the pathological state of osteoporosis. These findings underscore the significant impact of CD248 protein expression on the stemness and functional properties of TSPCs, highlighting its potential role in the pathophysiology of tendon healing and broader implications for tissue regeneration and repair.

The application of biomaterials in the treatment of RCT and OP has shown promising potential in enhancing healing outcomes.^[^ ^6^, ^55^ ^]^ In RCTs, biomaterials such as collagen scaffolds, hyaluronic acid, and bioactive glass are utilized to support tendon repair and regeneration.^[^ ^6^ ^]^ These materials provide a conducive environment for cellular growth, promote angiogenesis, and facilitate the integration of repaired tendons with bone. In the context of osteoporosis, biomaterials such as calcium phosphate cements and injectable bone substitutes are employed to enhance bone density and strength.^[^ ^56^, ^57^, ^58^ ^]^ These materials can be loaded with therapeutic agents such as bisphosphonates or osteoinductive growth factors to promote bone formation and inhibit resorption.^[^ ^28^ ^]^ The integration of biomaterials in both RCTs and OPs not only improves structural support but also accelerates the healing process by providing sustained release of bioactive molecules, thus addressing the underlying challenges of tissue degeneration and impaired healing associated with these conditions.

Liposomes are highly versatile and customizable spherical vesicles that have become pivotal in the field of biomaterials due to their ability to encapsulate and deliver a wide range of therapeutic agents.^[^ ^32^, ^33^, ^59^, ^60^ ^]^ In this study, si‐CD248‐loaded liposomes effectively restored the stemness of TSPCs and enhanced tendon‐bone healing in osteoporotic mice. We observed that the liposomes were biocompatible, as reported in the literature, and could be targeted to reduce the expression of our target proteins. In addition to these applications, liposomes are widely used in drug delivery, vaccine development, immunotherapy, and regenerative medicine. Their capacity for targeted delivery, controlled release, and biocompatibility makes them ideal for various therapeutic and diagnostic purposes. Ongoing advancements in liposome technology continue to improve their efficacy and safety, highlighting their significant potential in biomedical research and clinical applications.

CD248, also known as TEM1 or endosialin, is a transmembrane glycoprotein that plays a critical role in tissue remodeling and repair by binding to extracellular matrix components and promoting cell migration and attachment.^[^ ^61^, ^62^, ^63^ ^]^ Its expression, which increases in conditions such as cancer, infection, and fibrosis, is associated with disease severity and impacts key signaling pathways such as the PDGF‐BB and TGF‐β pathways.^[^ ^63^, ^64^ ^]^ Our findings suggest that CD248 drives TSPCs toward fibroblastic differentiation, consistent with our functional analysis results. These CD248^+^ TSPCs resemble more mature cells with lower stemness than regular TSPCs. This shift toward a more differentiated state suggests that targeting CD248 could enhance stem cell regenerative potential and improve tissue repair outcomes. Interestingly, CD248 has also been implicated in regulating the fibrotic response in tendon injuries by promoting ECM remodeling through FAK activation, suggesting that its modulation may not only affect TSPC behavior but also alter the composition and mechanical properties of the healing tissue. In addition, the FAK‐JAK‐STAT1 pathway plays a crucial role in regulating various cellular processes, including proliferation, migration, differentiation, and survival. In the context of stem cells, activation of the FAK‐JAK‐STAT1 signaling axis has significant implications for cell stemness, particularly affecting the balance between maintaining a stem cell state and differentiation. FAK is a critical signal mediator from the extracellular matrix (ECM). Enhanced interaction with the ECM via FAK activation can alter the stem cell niche, providing cues that promote differentiation.^[^ ^65^, ^66^, ^67^ ^]^ The STAT1 pathway directly influences the expression of several stemness markers. High levels of STAT1 activation are often correlated with reduced expression of key stemness genes such as OCT4, SOX2, and NANOG.^[^ ^68^, ^69^, ^70^ ^]^ Moreover, excessive activation of STAT1 can lead to a pro‐inflammatory environment that further disrupts the regenerative niche, creating conditions unfavorable for effective tissue repair. Mechanistically, CD248‐mediated activation of the FAK‐JAK‐STAT1 pathway alters the expression of downstream genes associated with apoptosis, proliferation, and migration. Specifically, activation of FAK leads to downstream phosphorylation of JAK and STAT1, which subsequently induces the expression of pro‐apoptotic genes such as Bax and Caspase‐3 while suppressing anti‐apoptotic molecules like Bcl‐2, contributing to increased apoptosis in CD248+ TSPCs. In addition, STAT1 regulates the expression of Cyclin D1 and p21, modulating cell cycle progression and reducing the proliferative capacity of TSPCs. These molecular changes collectively result in diminished stemness and regenerative potential, rendering CD248^+^ TSPCs less effective in contributing to tendon‐bone repair. Emerging evidence suggests that the FAK‐JAK‐STAT1 pathway interacts with other cellular pathways involved in tendon‐bone healing. For instance, the Wnt/β‐catenin pathway, which is crucial for tissue regeneration and stem cell self‐renewal, may synergize with FAK signaling to promote ECM remodeling and stem cell differentiation.^[^ ^71^, ^72^ ^]^ Activation of FAK has been shown to crosstalk with β‐catenin signaling by regulating β‐catenin nuclear translocation and enhancing its transcriptional activity, thereby influencing the expression of genes essential for ECM homeostasis and tissue repair.^[^ ^73^ ^]^ Additionally, the TGF‐β/Smad pathway, which plays a pivotal role in tissue fibrosis and inflammation, is influenced by changes in STAT1 activity. Enhanced STAT1 activity may suppress TGF‐β/Smad signaling, thereby modulating ECM remodeling and reducing fibrotic responses in tendon‐bone healing.^[^ ^74^ ^]^ Crosstalk between these pathways highlights the complexity of the signaling network regulating TSPC function and tendon‐bone healing, suggesting that targeting multiple pathways may yield synergistic therapeutic outcomes.

### 3.1 Limitations

While our study provides valuable insights into the role of CD248 and the FAK‐JAK‐STAT1 pathway in regulating TSPC stemness, several limitations must be acknowledged. First, our reliance on animal models and in vitro experiments may not fully replicate human physiology, necessitating clinical trials to validate the translational applicability of our findings. Second, the long‐term effects and potential side effects of si‐CD248‐loaded liposome treatment have not been extensively explored, highlighting the need for further investigation into the sustained benefits and possible adverse effects of these treatments. Finally, our focus on the FAK‐JAK‐STAT1 pathway, without considering other signaling pathways and environmental factors influencing TSPC behavior, calls for a more comprehensive analysis in future studies to fully understand the molecular mechanisms governing tendon‐bone healing and stem cell function.

## 4 Conclusion

In conclusion, our study demonstrated that CD248 plays a critical role in modulating the stemness of TSPCs under osteoporotic conditions. We showed that CD248^+^ TSPCs exhibit reduced proliferative capacity, increased apoptosis, and impaired migratory ability, primarily due to activation of the FAK‐JAK‐STAT1 signaling pathway. By using si‐CD248‐loaded liposomes, we successfully inhibited CD248 expression, thereby restoring TSPC stemness and enhancing tendon‐bone healing in osteoporotic mice. These findings suggest that targeting CD248 can effectively enhance the regenerative potential of TSPCs, suggesting that targeting CD248 is a promising therapeutic strategy for improving tendon repair in osteoporotic patients. Despite these promising results, further studies are needed to validate these findings in human models and to explore the long‐term safety and efficacy of CD248‐targeted therapies. This research paves the way for new avenues in regenerative medicine, particularly in the treatment of tendon injuries and other conditions involving compromised tissue healing.

## 5 Experimental Section

### Animal Modeling and Ethics

Animal experiments were approved by the Ethics Committee for Animal Experiments of Nanjing University of Chinese Medicine, ethical approval number: (A220606). Osteoporotic mice were induced by removing the ovaries of female mice for 3 months. Bone density indices were examined by CT, as reported in a previous study.^[^ ^34^ ^]^ The creation of a rotator cuff tear model in osteoporotic mice involves several critical steps: Mice are anesthetized and placed in a supine position, and the forelimbs are abducted using a needle. A 2 cm ventral incision was made for access to the rotator cuff. The shoulder was positioned to access the proximal humerus, with the forelimb extended, adducted, and externally rotated. The supraspinatus tendon was exposed and elevated. Suture attachment was made to the tendon using a modified Kessler suture pattern. Materials such as type I collagen hydrogels encapsulating functionalized liposomes were placed at the interface of the tendon and humeral head. Finally, bone tunnels were created in the humeral head to secure the tendon.

### Single‐Cell Suspensions, Library Construction, and Sequencing

Three months after the ovary removal procedures, tendon samples were promptly harvested from the mice. Single‐cell suspensions were then created via mechanical dissociation and enzymatic digestion, with concentration adjustments as necessary. For library preparation, the Chromium Single Cell 3′ Library & Gel Bead Kit v2, Chromium Single Cell 3′ Chip Kit v2, and Chromium i7 Multiplex Kit were utilized according to the manufacturer's guidelines. The prepared libraries were subsequently sequenced using the Illumina NovaSeq 6000 system.

### Single‐Cell RNA‐Seq (scRNA‐Seq) Data Processing

The CellRanger‐4.0.0 R package was employed to generate raw fastq files using the “cellranger‐arc mkfastq” function.^[^ ^35^, ^36^ ^]^ These raw fastq files were then aligned to the reference genome, filtered, and subjected to barcode counting and unique molecular identifier counting to produce single‐cell gene expression profiles through the “cellranger count” function. To address any potential biases, the “cellranger aggr” function was used to aggregate gene expression counts across all samples, normalizing these counts to a consistent sequencing depth and recalculating the feature‐barcode single‐cell gene expression matrices. The raw gene expression data were imported into R (version 4.2.0) for analysis with the Seurat R package (version 4.3.0). Quality control was performed using the Doublet Finder R package (version 2.0.3) with specific thresholds: 1) 300 < nFeature < 7500; 2) 500 < nCount < 100 000; 3) mitochondrial gene expression ≤ 25% of total gene expression; and 4) erythroid gene expression ≤ 5% of total gene expression. The Harmony algorithm (version 0.1.1) was employed for batch effect correction. The dimensionality reduction parameter (dim) was set to 30, while the resolution parameter was set to 1.2. The “Normalize Data” and “Scale Data” functions from Seurat were used for logarithmic normalization and linear regression, respectively. The “Find Variable” function identified the top 2000 most variable genes for principal component analysis. The top 30 principal components, with a resolution of 1.0, were used in the “Find Clusters” function to identify distinct cell clusters.

### Determination of Cell Subtypes

Cell clusters were initially identified using the “Find Clusters” and “Find Neighbors” functions in Seurat, with a default resolution of 0.8. Clusters were then annotated based on the average gene expression of canonical markers.^[^ ^37^, ^38^ ^]^ The “Find All Markers” function in Seurat was used for the Wilcoxon rank‐sum test for DEGs across cellular clusters, with the min.pct and min.diff.pct parameters set to 0.25 and the logfc.threshold set to 0.25.

### Pathway Enrichment Analysis

Enrichment analyses of DEGs in various cell types were conducted using Gene Ontology (GO) and gene set enrichment analysis (GSEA) tools^[^ ^39^ ^]^ with the Cluster Profiler R package (version 4.6.0). The significance of GO terms was determined based on an adjusted *p* < 0.05.

### Gene Set Scoring

To score gene sets in the scRNA‐seq data, we utilized the “AUCell” method from the irGSEA R package, with gene sets sourced from previously published articles and available at https://www.gsea‐msigdb.org/gsea/msigdb and https://reactome.org/.

### Developmental Trajectory Inference

Cellular stemness was assessed using the CytoTRACE R package (version 0.3.3),^[^ ^40^ ^]^ enabling inference of temporal cell differentiation progression. Cell differentiation trajectories were constructed with the Monocle R package (version 2.24.0), employing the uniform manifold approximation and projection (UMAP) method for dimension reduction and subsequent visualization via the “PLOT‐CELL‐TRAJECTORY” function. The different cell subpopulations were then sorted based on pseudotime order, with genes showing synchronized changes along the pseudotime trajectory identified and represented in a pseudotime heatmap. The Slingshot R package (version 2.6.0) was used to infer cell lineages and pseudotime, utilizing a clustering‐based minimum spanning tree to identify lineage structures and simultaneous principal curves to fit branch curves. The “getCurves” function obtained smoothed trajectory curves.

### Cell–Cell Communication Analysis

The CellChat R package (version 1.6.1) was used to infer complex cell‒cell interactions and establish regulatory networks based on ligand‒receptor levels. The “net Visual DiffInteraction” function represented differences in communication strength between cells, while the “Identify Communication Patterns” function estimated the number of communication patterns, with a significance threshold of *p* < 0.05.

### Micro‐CT

The anesthetized mice were placed on the sample stage of the MicroCT scanner (Skyscan 1176). The mouse was secured using a specialized fixation device so that its femur was in the optimal scanning position. The MicroCT scanner started to perform a tomography scan of the sample. The reconstructed 3D images were analyzed for bone density using professional image analysis software (CT‐Analyzer, CTVox).

### si‐CD248‐Loaded Liposome Preparation and Characterization

The liposome components, comprising C12‐200, cholesterol, DSPC, and mPEG‐DMG, were dissolved in ethanol to achieve a molar ratio of 50:38.5:10:1.5.^[^ ^29^, ^41^ ^]^ This mixture was then combined with either si‐CD248 or si‐Ctrl, both of which were dissolved in citrate buffer (10 mm or 50 mm, pH  =  3), through vortexing. Ultrafiltration centrifugation was employed to remove any free siRNA. The encapsulation efficiency was determined using a Quant‐iT RiboGreen RNA assay kit (Molecular Probes, UK) and measured with a spectrofluorometer set to an excitation wavelength of 480 nm and an emission wavelength of 520 nm. The resulting liposomes were diluted in PBS before use. To further assess entrapment efficiency, the RiboGreen assay was utilized. Additionally, the hydrodynamic diameter, zeta potential, polydispersity index, and stability of the liposomes were quantified using dynamic light scattering (Malvern Zetasizer, Nano‐ZS, UK), and their morphology was observed via transmission electron microscopy (TEM). Particle size and visualization were performed via nanoparticle tracking analysis (NTA). Biocompatibility is reflected by tissue sections of multiple organs.

The in vitro siRNA release profile was evaluated using a dialysis method. Liposome suspensions were placed in dialysis bags (molecular weight cutoff: 10 kDa), immersed in phosphate‐buffered saline (PBS, pH 7.4), and incubated at 37 °C under gentle shaking. At predetermined time intervals (0, 1, 3, 6, 12, 24, 48, and 72 h), aliquots of the dialysis medium were collected and replenished with an equal volume of fresh PBS. The released siRNA concentration was measured using the Quant‐iT RiboGreen RNA assay kit.

Subsequently, fluorescence tracking assays were performed to examine liposome‐TSPC interactions in vitro. Liposomes were labeled using DiI fluorescent dye (Beyotime Biotechnology, China). TSPCs were cultured on confocal dishes and incubated with DiI‐labeled liposomes at 37 °C for intervals of 12 h. Post‐incubation, cells were washed thoroughly with PBS, fixed with 4% paraformaldehyde, and stained with DAPI for nuclear visualization. Confocal laser scanning microscopy (CLSM, Leica Microsystems, Germany) revealed progressive internalization of DiI‐labeled liposomes by TSPCs.

For in vivo evaluation, fluorescently labeled liposomes were intravenously injected into the tail veins of nude mice. Fluorescence distribution was monitored at different time points (12 h, 24 h, 48 h, 5 d, and 10 d post‐injection) using an in vivo imaging system (IVIS Lumina Series III, PerkinElmer, USA).

### Gait Analysis

The locomotive performance of the mice was carefully evaluated using the CatWalk XT gait assessment system (CatWalk XT; Noldus, Netherlands).^[^ ^42^, ^43^ ^]^ For mice in the RCT model group and the liposome treatment group, surgery was limited to the right forefoot, and the contralateral limb was left intact. This approach is intended to avoid postural changes that may occur when mice are trying to maintain balance. Such postural changes may affect the ability of mice to discriminate between pairs of hind feet. However, the limbs of the control mice were not subjected to any surgical alterations.

To acclimate the mice to the experimental conditions, an intensive training schedule was applied, demanding that the mice move through a confined passage from one point to another over a light‐emitting glass runway. The focus was on ensuring a continuous stride without significant stops, lasting no less than 7 days. Images of the mice left by the mice were captured via a built‐in illumination footprint diffraction method. Each impression offered insight into not only the size but also the distribution of pressure from the mouse's foot. The brightness levels of the emitted light correlated with the pressure intensity. High‐resolution cameras were utilized to capture the footprints precisely, which were later analyzed automatically using Catwalk XT 10.0 software.

### Statistical Analysis

All experiments in this study were conducted at least three times. The data were analyzed using GraphPad Prism 7.0 (GraphPad Software, La Jolla, CA) and are presented as the mean±SD. The Mann–Whitney U test, nonparametric Student's *t*‐test, one/two‐way analysis of variance (ANOVA), or analysis of variance followed by post hoc Bonferroni correction were applied to evaluate the differences between groups based on the normality of the distribution of the data. *p* < 0.05 was regarded as significant. For single‐cell analysis, R software and Python software were used for database data analysis. All *p*‐values are two‐tailed, with values less than 0.05 indicating statistical significance. Values less than 0.001 were considered highly significant, while those less than 0.0001 were considered extremely significant.

## Conflict of Interest

The authors declare no conflict of interest.

## Author Contributions

Y.Q., J.Z., Y.H., and H.Q. contributed equally to this work and shared the first authorship. Y.Q., J.Z., and Y.H. performed the investigation and formal analysis, developed methodology, and wrote, reviewed, and edited the final draft. P.Q., B.S., H.H., H.Q., and Y.O. developed the methodology and performed formal analysis and investigation. C.K. and Q.Y. performed formal analysis and investigation. F.X., Z.L., X.W., and Q.W. conceptualized and visualized the study and wrote, reviewed, and edited the final draft.

## Supporting information



Supporting Information

Supplemental Table 1

## Data Availability

The data that support the findings of this study are available from the corresponding author upon reasonable request.
