# Supplementary material for: Bioactive siRNA‐Based Liposomes Promoted Tendon‐Bone Healing in Osteoporotic Mice by Recovering the Stemness of CD248+ TSPCs
Source: Adv Sci (Weinh). 2025 Jun 19;12(34):e09883. doi: 10.1002/advs.202509883 (PMC12442593; doi:10.1002/advs.202509883)
Supplement: Supplementary file 1 — Supporting Information [file ADVS-12-e09883-s001.docx]

**Bioactive siRNA-based Liposomes Promoted Tendon-Bone Healing in Osteoporotic Mice by Recovering the Stemness of CD248^+^ TSPCs**

**Supplemental files:**

**Supplemental methods:**

**Cell culture and treatment**

TSPCs were sorted from tendon tissues throughout the body of mice using flow cytometry. A specialized stem cell culture medium was used for culture. The TSPCs were placed in a 37 °C incubator containing 5% carbon dioxide. Ontuxizumab (10 µg/mL) and siRNA (20 nM) were used in the cell experiments.

**CD248 overexpression**

To generate a stable CD248-overexpressing TSPC cell line (CD248+ TSPCs), we used a plasmid transfection protocol. The full-length Cd248 DNA fragment (PubMed Gene ID 57124) was cloned and inserted into the pEX-5-CMV™ expression vector (GenScript, Shanghai). Mouse TSPCs were seeded in a 6-well plate at a density of 2 × 105 cells per well and cultured overnight to 60–70% confluence. The next day, the CD248 expression plasmid (1 µg/mL) was transfected into the cells using Lipofectamine 3000 (Thermo Fisher Scientific) according to the manufacturer’s instructions. The DNA-Lipofectamine complexes were prepared by mixing the plasmid DNA with Lipofectamine 3000 reagent in Opti-MEM and incubating for 15-20 minutes at room temperature. The mixture was then added dropwise to the cells and incubated for 6 hours, followed by replacement with fresh culture medium. To establish stable cell lines, cells were selected with G418 (400 µg/mL) for 2 weeks. Resistant colonies were expanded, and CD248 overexpression was confirmed by western blot analysis.

**Proliferation and migration experiments**

***EdU test***: To assess the proliferative capacity of TSPCs, we conducted an EdU incorporation assay using a Click-iT EdU Imaging Kit (Thermo Fisher Scientific) following the manufacturer's instructions. TSPCs were seeded in a 24-well plate at a density of 1 x 10^4 cells per well and allowed to adhere overnight. The following day, the cells were incubated with 10 µM EdU for 2 hours at 37°C to allow for incorporation into the newly synthesized DNA. After incubation, the cells were fixed with 4% paraformaldehyde for 15 minutes at room temperature and permeabilized with 0.5% Triton X-100 in PBS for 20 minutes. The cells were then incubated with the Click-iT reaction cocktail for 30 minutes in the dark, which facilitated the fluorescent labeling of EdU. Nuclei were counterstained with DAPI for 5 minutes, and the cells were imaged using a fluorescence microscope. The percentage of EdU-positive cells was quantified by counting the number of EdU-positive nuclei relative to the total number of DAPI-stained nuclei.

***Wound-Healing Assay***[33]: TSPC cells were inoculated in 6-well plates, and when the cell confluence reached 100%, a straight line was drawn at the bottom of the cell culture plate using a sterile pipette suction head, and the shed cells were washed and cultured in base medium containing 1% fetal bovine serum.

**Apoptosis Staining**

An Annexin V-FITC apoptosis assay kit (Beyotime Biotechnology, Shanghai, China) was used to detect the percentage of apoptotic cells. The experimental procedure was carried out according to the kit instructions.

**Flow Cytometry**

***Cell sorting***

To isolate TSPCs from mouse tendon tissue, we employed a flow cytometry-based cell sorting protocol. Tendon tissues were carefully dissected from the hind limbs of euthanized mice under sterile conditions. The tissues were then minced into small pieces and subjected to enzymatic digestion in a solution containing collagenase type I (2 mg/mL) and dispase (2 mg/mL) in DMEM for 2-3 hours at 37°C with gentle agitation. The resulting cell suspension was filtered through a 70 µm cell strainer to remove debris and washed twice with PBS containing 2% FBS. The cells were then resuspended in PBS/2% FBS and incubated with fluorochrome-conjugated antibodies against CD45, CD31, and Sca-1 for 30 minutes on ice in the dark. After incubation, the cells were washed twice with PBS/2% FBS and resuspended at a concentration of 1 × 106 cells/mL. The labeled cell suspension was then subjected to flow cytometric sorting using a FACSAria III cell sorter (BD Biosciences). TSPCs were identified as CD45-CD31-Sca-1+ cells and were sorted into sterile tubes containing DMEM/10% FBS. CD248+ TSPCs were identified as CD45-CD31-Sca-1+CD248+ cells from osteoporotic mice. The purity and viability of the sorted cells were assessed via flow cytometry, and the isolated TSPCs were subsequently cultured under standard conditions for subsequent experiments. This method ensures the precise isolation of TSPCs, enabling further functional and molecular studies.

***Apoptosis cytometry***

To assess apoptosis in TSPCs, we used Annexin V-FITC/propidium iodide (PI) dual staining combined with flow cytometry analysis. Cultured TSPCs were harvested by trypsinization and gently pipetted to form a single-cell suspension. The cells were then washed twice with cold PBS and resuspended in Annexin V binding buffer at a concentration of approximately 1 x 106 cells/mL. Subsequently, 5 µL of Annexin V-FITC and 5 µL of PI were added to the cell suspension, followed by gentle mixing and incubation in the dark at room temperature for 10-15 minutes. After staining, 400 µL of Annexin V binding buffer was added to each sample, and the samples were immediately analyzed using a flow cytometer (BD) with a 488 nm laser for excitation of FITC and PI, and fluorescence signals were collected in the FL1 and FL2 channels, respectively. Data analysis was performed using flow cytometry software (such as FlowJo), which categorizes the cells into four populations: Annexin V-FITC negative/PI negative (viable cells), Annexin V-FITC positive/PI negative (early apoptotic cells), Annexin V-FITC positive/PI positive (late apoptotic or necrotic cells), and Annexin V-FITC negative/PI positive (dead cells). This method enables quantitative assessment of apoptosis in TSPCs under various experimental conditions, providing insights into the mechanisms of cell survival and death.

***Cell cycle***

To analyze the cell cycle distribution of TSPCs, cultured TSPCs were first collected after digestion with trypsin. The cells were suspended in cold PBS, and the cell precipitate was collected by centrifugation. The cell precipitates were fixed with 70% cold ethanol overnight. After fixation, the cells were washed twice with PBS to remove the ethanol residue. Subsequently, the cells were suspended in PBS solution containing 50 µg/mL RNase A and 100 µg/mL propidium iodide (PI) and incubated at 37°C for 30 min in the dark.

**Western blot (WB)**

Western blotting was performed as previously described [40]. Lysis buffer for WB/IP assays (Yeasen, Shanghai, China) supplemented with protease inhibitors was used to lyse the cells to obtain total protein. After cracking on ice for 15 min, the samples were centrifuged at 10000 rpm at 4°C for 15 min to obtain the serum. The total protein concentration was determined by a BCA protein quantification kit (Yeasen, Shanghai, China). Thirty micrograms of protein was electrophoretically separated on a 10% SDS‒PAGE gel, transferred to a PVDF membrane, enclosed in 5% skim milk powder at room temperature for 1 h, and then incubated with primary and secondary antibodies. Primary antibodies against CD248, FAK, p-FAK, Bcl, Bax, Caspase 3, CD31, JAK1, p-JAK1, STAT1, p-STAT1, GAPDH, and tubulin (Abcam, US, or Affinity, China, 1:1000) were incubated at 4°C overnight, and secondary antibodies conjugated to horseradish peroxidase were incubated with the membranes at room temperature for 1 hour. Then, Image Lab was used to detect protein expression, and ImageJ was used to analyze protein gray values.

**Reverse Transcription Quantitative Polymerase Chain Reaction (RT-qPCR)**

Total RNA was extracted using Trizol reagent (Tiangen, China) following the manufacturer's protocol and reverse-transcribed into cDNA with RT Super Mix (Tiangen, China). Subsequently, real-time PCR was performed using the SYBR Green Real-Time Fluorescent Quantitative Kit (Tiangen, China) on a quantitative PCR system (Mastercycler ep realplex, Eppendorf, Germany) to determine gene expression levels. The primer sequences used are listed in Table S1. Finally, the relative mRNA expression levels were calculated using the 2−ΔΔCt method.

**siRNA construction**

To knock down CD248 expression in mouse TSPCs, we employed a siRNA transfection protocol using Lipofectamine RNAiMAX (Thermo Fisher Scientific) according to the manufacturer's instructions. TSPCs were seeded in a 6-well plate at a density of 2 × 105 cells per well and cultured overnight to 60–70% confluence. The next day, the siRNA targeting CD248 (si-CD248) and a nontargeting control siRNA (si-Ctrl) were diluted in Opti-MEM reduced serum medium to a final concentration of 20 nM. Lipofectamine RNAiMAX reagent was also diluted in Opti-MEM. The diluted siRNA and Lipofectamine RNAiMAX were combined and incubated for 20 minutes at room temperature to form siRNA-Lipofectamine complexes. These complexes were then added dropwise to the cells, followed by gentle rocking to ensure even distribution. The cells were incubated with the transfection mixture for 6 hours, after which the medium was replaced with fresh culture medium. Transfection efficiency and CD248 knockdown were verified 48 hours posttransfection by western blot analysis. This method effectively introduces siRNA into TSPCs, leading to the targeted knockdown of CD248 for subsequent functional studies. The sequences of the siRNAs used were as follows: 5′‐GGCUUCGAGUGUUAUUGUAUU‐3′ for CD248 and 5′‐UUCUCCGAACGUGUCACGUU‐3′ for the negative control siRNA-si-Ctrl (GenePharma, Shanghai, China).

**Histological Examination**

To assess tendon-bone healing and regeneration, histological examination was conducted on specimens from mice subjected to different treatments. Specimens were fixed in paraformaldehyde, decalcified in EDTA decalcifying solution, embedded in paraffin after decalcification, and sectioned into 5 μm slices using a microtome for subsequent H&E staining/Masson Staining/Fuchsin Solid Green Staining/Immunofluorescence staining. For immunohistochemistry (IHC) staining, the sections were deparaffinized and blocked with 3% H2O2. The plates were then incubated overnight.

at 4°C with the appropriate primary antibodies. After removal of the primary antibody, the sections were incubated with secondary antibodies for 60 min at room temperature. Finally, the stained sections were developed using diaminobenzidine solution and counterstained with hematoxylin.

**Biomechanical Testing**

The failure load and ultimate strength of the samples were evaluated using a materials testing system. The specimens were secured in fixtures and subjected to uniaxial tensile testing with a 60° abduction angle to approximate the anatomical position of the specimens. Tension was applied at a rate of 1 millimeter per minute until failure.

**Supplemental Figures:**


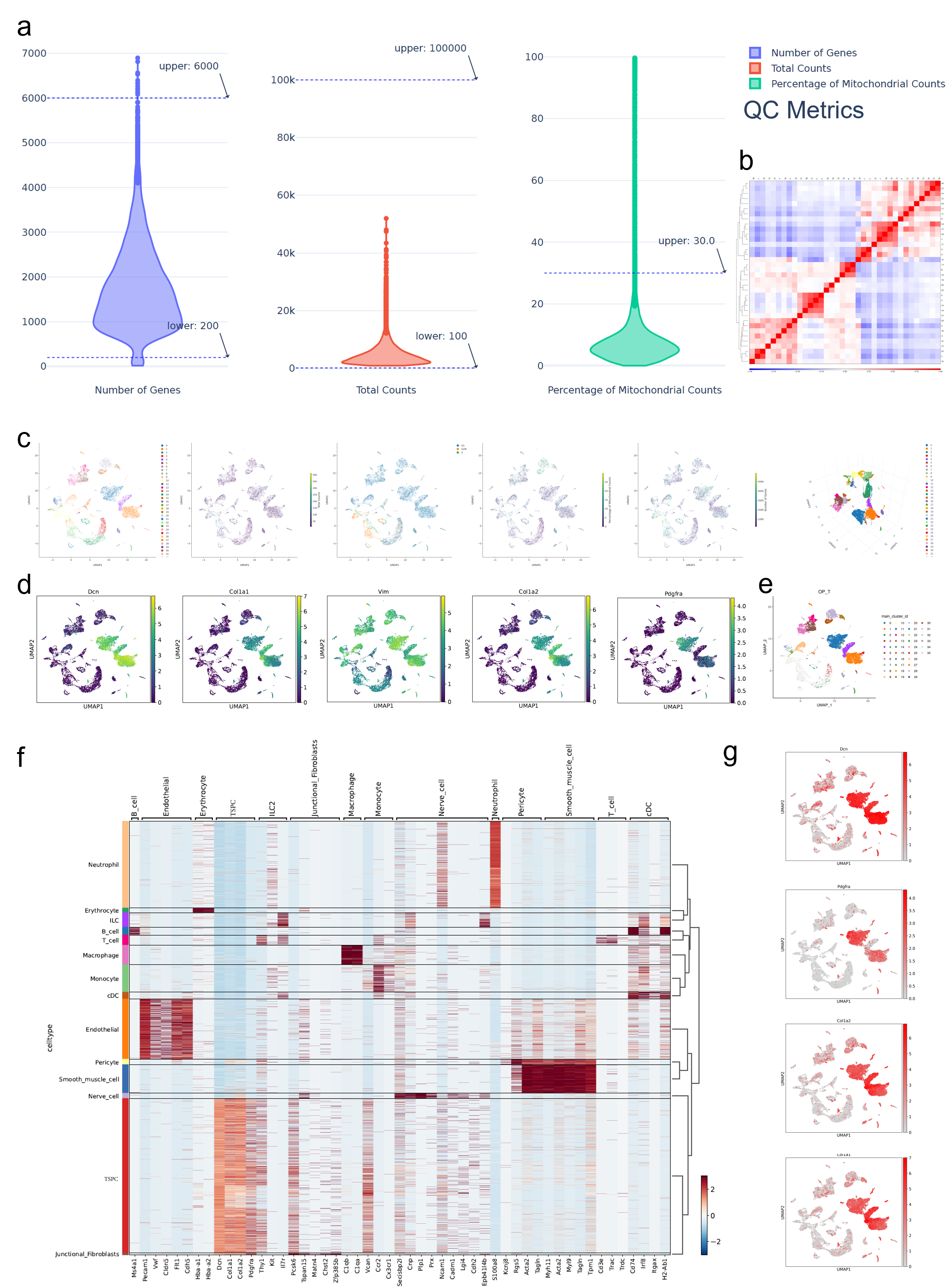


**Supplementary Figure 1. Quality Control and Marker Gene Analysis of Single-cell RNA Sequencing Data**

**a. Violin plots of QC metrics**
These violin plots display the quality control metrics of the single-cell RNA sequencing data, including the number of genes detected per cell, total counts per cell, and the percentage of mitochondrial counts. The upper and lower thresholds for each metric are indicated.

**b. Heatmap of cell-to-cell correlation**
This heatmap illustrates the correlation between cells based on their gene expression profiles, highlighting the overall similarity and clustering of the cells.

**c. UMAP plots of other parameters**

**d. UMAP plot of differentially expressed genes**
The **UMAP** plot displays the expression patterns of differentially expressed genes across various cell types. Each row represents a gene, and each column represents a cell type, with colors indicating expression levels.

**e. UMAP plot of cell type distribution in 3D**
This UMAP plot highlights the distribution of different cell types identified in the single-cell RNA sequencing data, with each color representing a distinct cell type.

**c. Heatmap of gene expression profiles across cell types**
The heatmap shows the gene expression profiles of the different **cell types**, highlighting the distinct transcriptional signatures associated with each subcluster.

**g. Feature plots of additional marker genes**
These feature plots show the expression levels of additional marker genes (e.g., Thbs2, Bgn, and Comp) across the UMAP space, providing further validation of cell type identification.


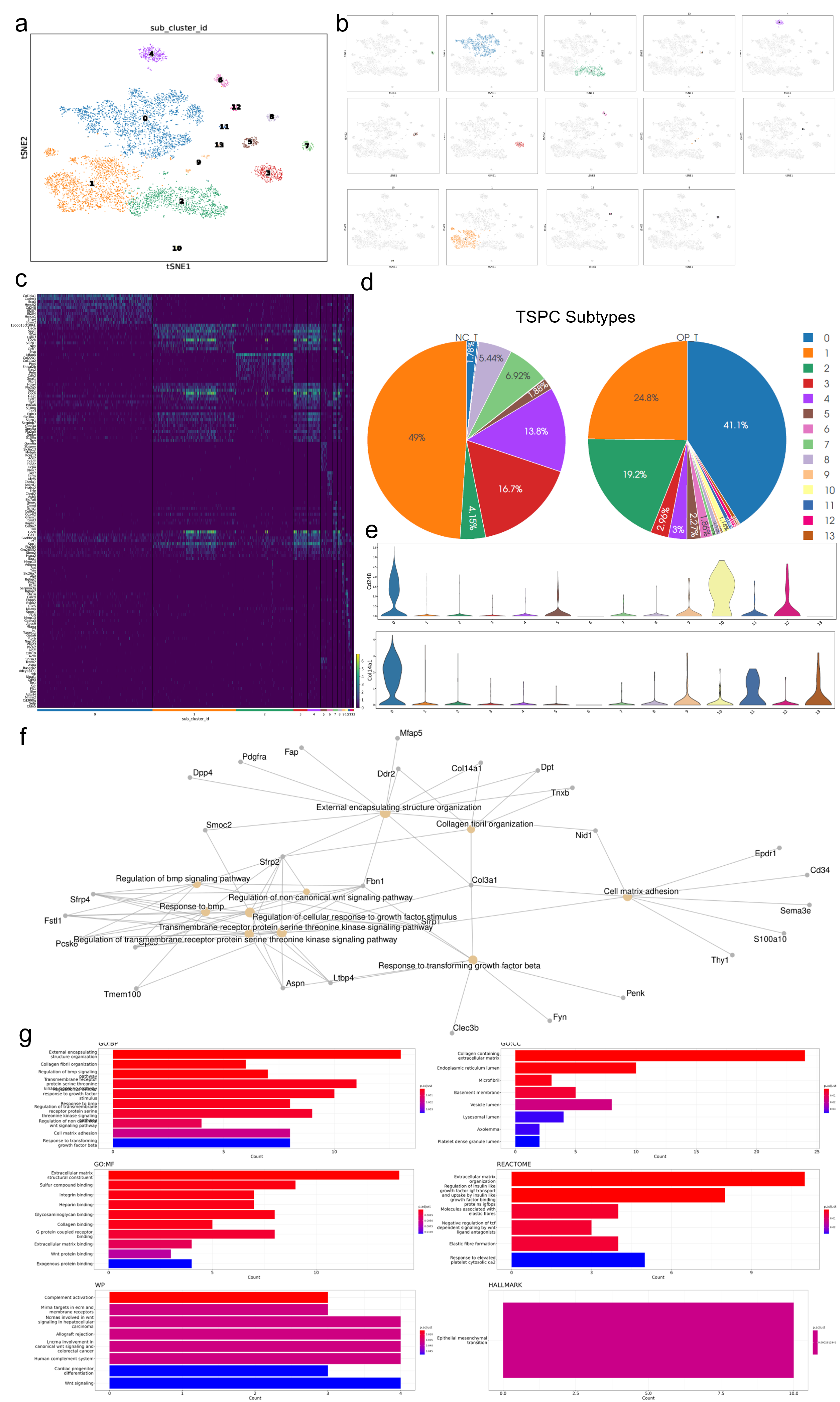


**Supplementary Figure 2. Detailed Subcluster Analysis of Single-cell RNA Sequencing Data**

**a. UMAP plots of additional gene expression**
These UMAP plots illustrate the expression levels of additional marker genes (e.g., gene1, gene2, gene3) across different subclusters, further validating the identity and heterogeneity of the subclusters.

**b. Feature plots of subclusters in the UMAP plot**

**c. Heatmap of cell-to-cell correlation within subclusters**
This heatmap shows the correlation between cells within each subcluster, indicating the internal consistency and similarity of gene expression profiles within subclusters.

**d. Bar plot of subcluster proportions between the control and osteoporotic groups**
This bar plot quantifies the proportions of each subcluster in the control group (NC_T) and the osteoporotic model group (OP_T), highlighting the significant expansion of TSPC-0 in the osteoporotic group.

**e. Violin plots of pathway activity scores**
These violin plots display the distribution of pathway activity scores across different subclusters, providing insights into the functional differences among subclusters.

**f. Network diagram of pathway enrichment in subclusters**
This network diagram presents the enrichment of various signaling pathways in different subclusters. The size of the dots represents the fraction of cells involved in each pathway, and the color intensity indicates the mean expression level.

**f. Heatmap of DEGs in subclusters**
This heatmap depicts the DEGs across various subclusters, with each row representing a gene and each column representing a subcluster. The colors indicate the relative expression levels.

**g. Enrichment analysis histogram of TSPCs**


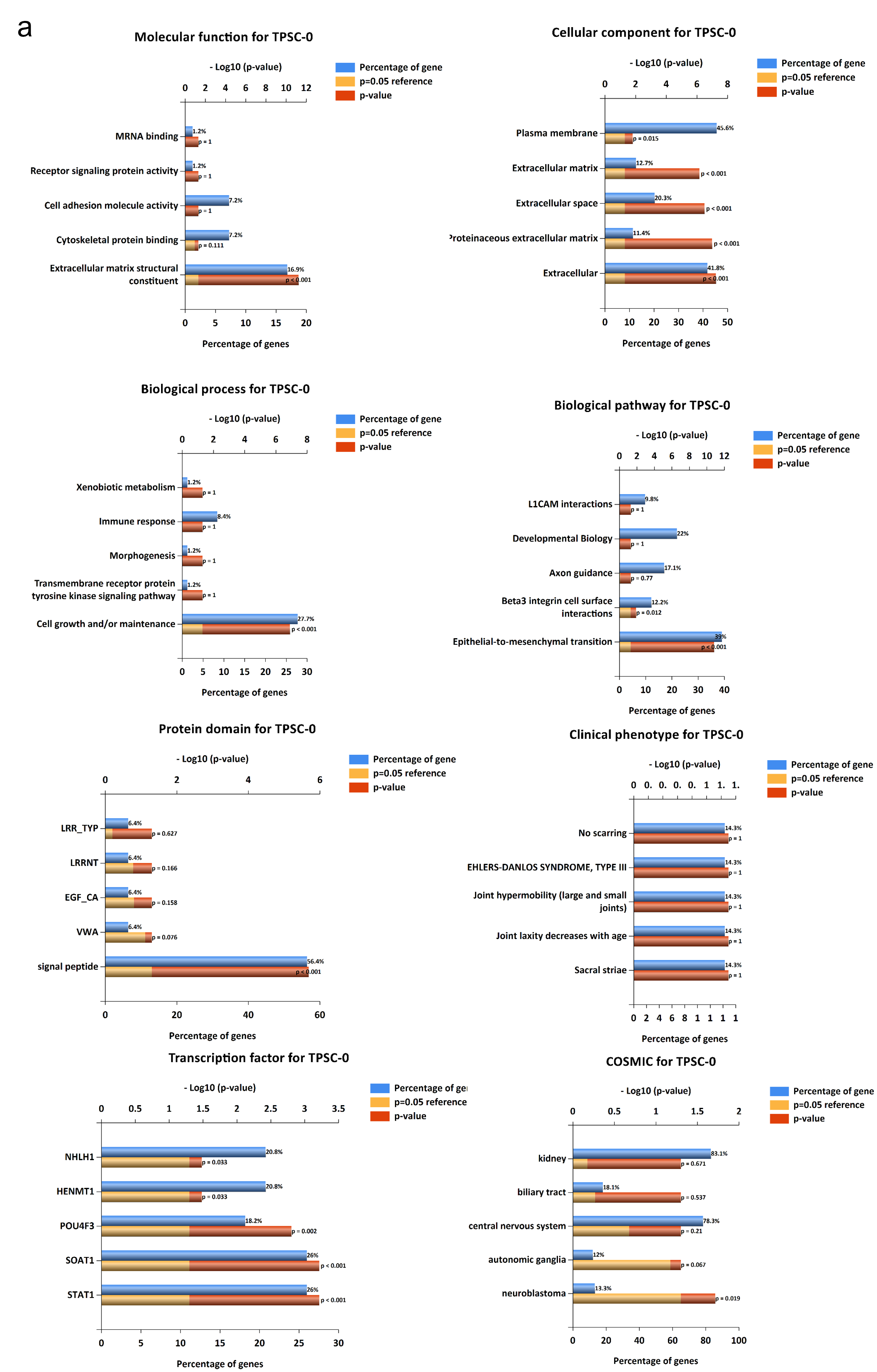


**Supplementary Figure 3. Functional enrichment analysis of the TSPC-0 subcluster**

**a. Molecular function enrichment for TSPC-0**
This bar plot illustrates the enrichment of various molecular functions within the TSPC-0 subcluster. The significant categories included extracellular matrix structural constituents, cytoskeletal protein binding, and receptor signaling protein activity.

**Cellular component enrichment for TSPC-0**
This bar plot shows the enrichment of cellular components within the TSPC-0 subcluster. Notable components include the extracellular matrix, extracellular space, and plasma membrane.

**Biological process enrichment for TSPC-0**
This bar plot presents the enrichment of biological processes in the TSPC-0 subcluster. The significant processes included cell growth and/or maintenance, the immune response, and the transmembrane receptor protein tyrosine kinase signaling pathway.

**Biological pathway enrichment for TSPC-0**
This bar plot highlights the enriched biological pathways in the TSPC-0 subcluster. Key pathways include epithelial-to-mesenchymal transition, Beta3 integrin cell surface interactions, and axon guidance.

**Protein domain enrichment for TSPC-0**
This bar plot shows the enrichment of specific protein domains within the TSPC-0 subcluster. The significant domains included the signal peptide, VWA, and LRRNT domains.

**Clinical phenotype associations for TSPC-0**
This bar plot depicts the associations between TSPC-0 and various clinical phenotypes. Notable phenotypes include sacral striae, joint laxity decreases with age, and Ehlers‒Danlos syndrome type III.

**Transcription factor enrichment for TSPC-0**
This bar plot presents the enrichment of transcription factors within the TSPC-0 subcluster. The significant transcription factors included STAT1, SOAT1, and POU3F3.

**COSMIC gene enrichment for TSPC-0**
This bar plot illustrates the enrichment of genes from the Catalog Of Somatic Mutations In Cancer (COSMIC) within the TSPC-0 subcluster. The significant categories included neuroblastoma, autonomic ganglia, and the central nervous system.


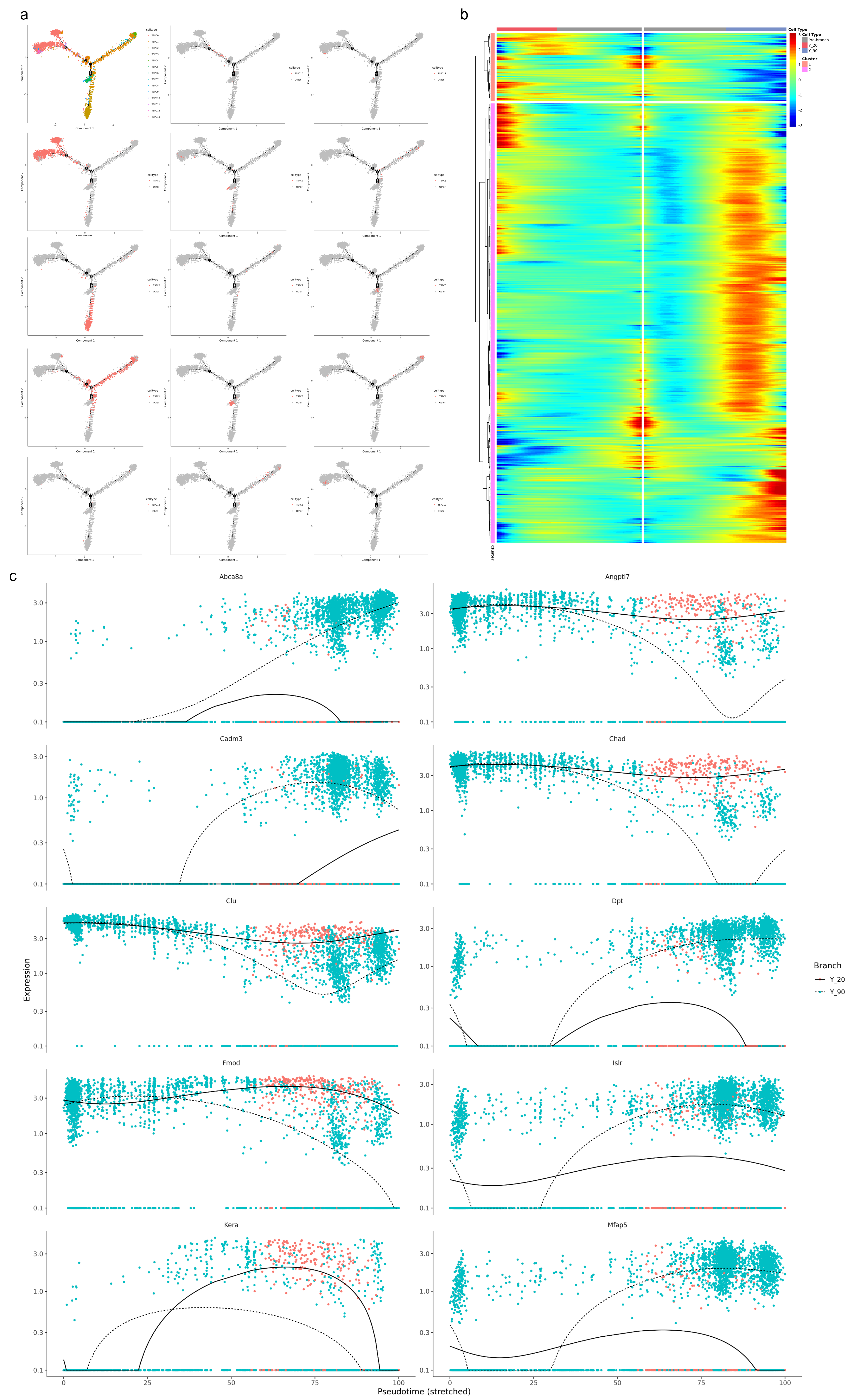


**Supplementary Figure 4. Pseudotime analysis of gene expression dynamics**

**a. Pseudotime trajectory analysis colored by various attributes**
This panel shows the pseudotime trajectory of cells, colored by different attributes such as cell type, sample group, and gene expression levels. Each plot highlights the progression of cells through pseudotime and the distribution of these attributes along the trajectory.

**b. Heatmap of dynamic gene expression along pseudotime**
This heatmap illustrates the expression patterns of dynamic genes across the pseudotime trajectory. Each row represents a gene, and each column represents a position along pseudotime, with colors indicating expression levels. The heatmap reveals the temporal changes in gene expression as cells transition through different states.

**c. Scatter plots of selected gene expression levels over pseudotime**
These scatter plots display the expression levels of selected genes (e.g., Alcam, Cadm3, Clu, Fmod, Kera, Angptl7, Chad, Dpt, Islr, and Mfap5) over pseudotime. Each dot represents a cell, colored by branch, with a trend line indicating the overall expression trend along pseudotime.


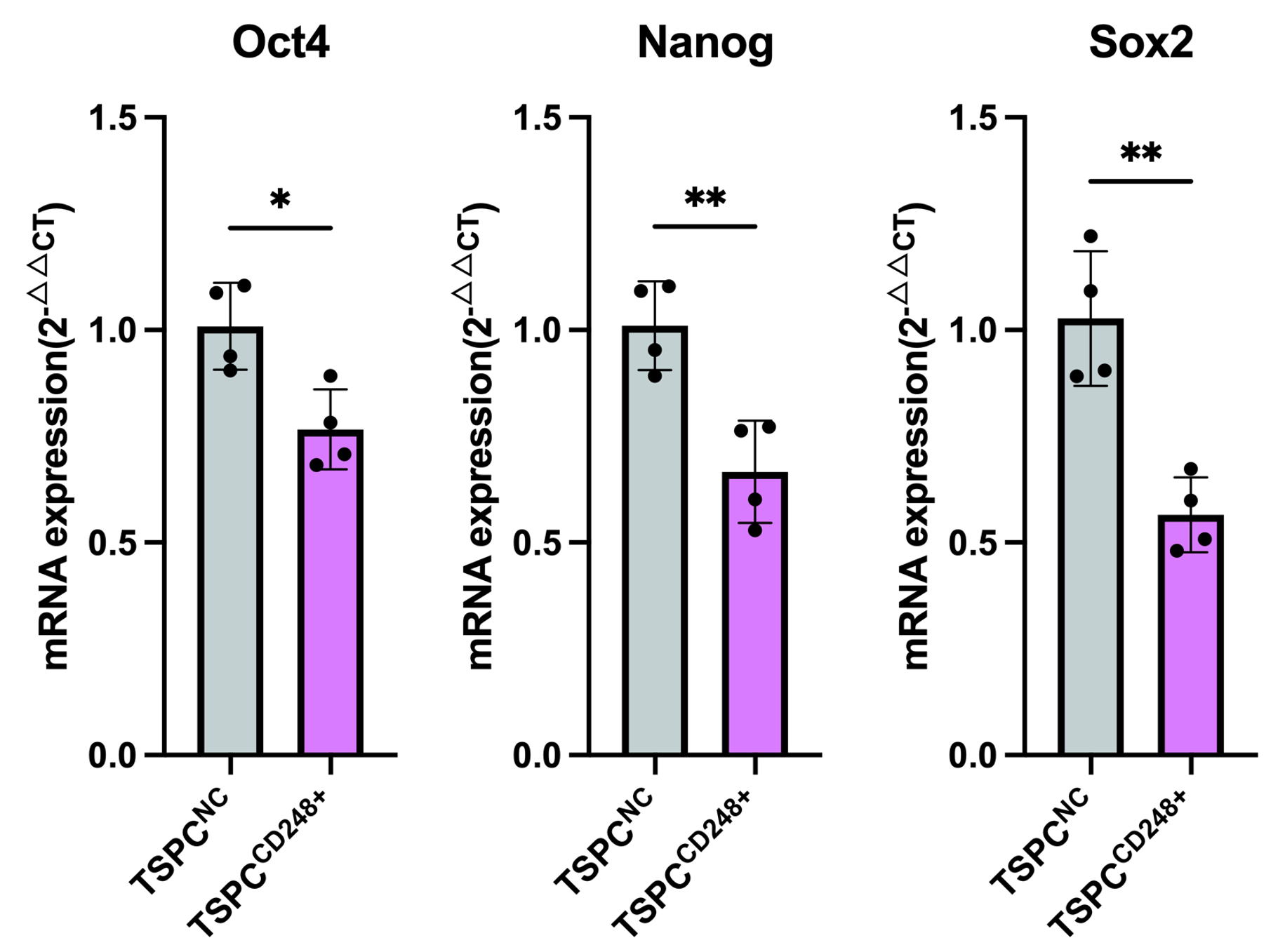


**Supplementary Figure 5. Validation of stem markers of TSPCs**

Quantification of the qPCR results. The data are presented as the mean ± SD. *P < 0.05, **P < 0.01.


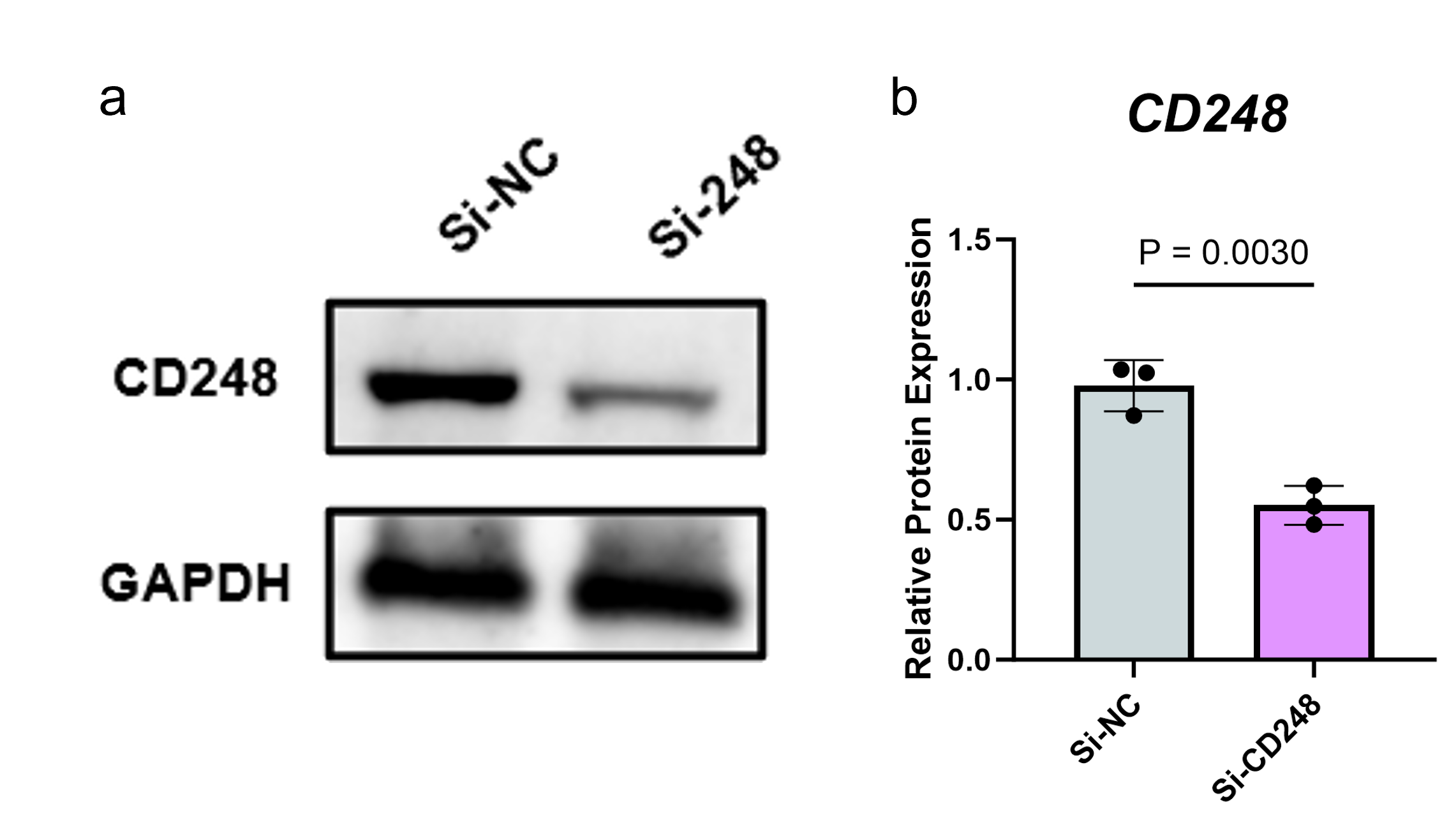
**Supplementary Figure 6. Validation of CD248 knockout**

1. WB results
2. Quantification of the WB results. The data are presented as the mean ± SD. *P < 0.05, ***P < 0.001, ****P < 0.0001.


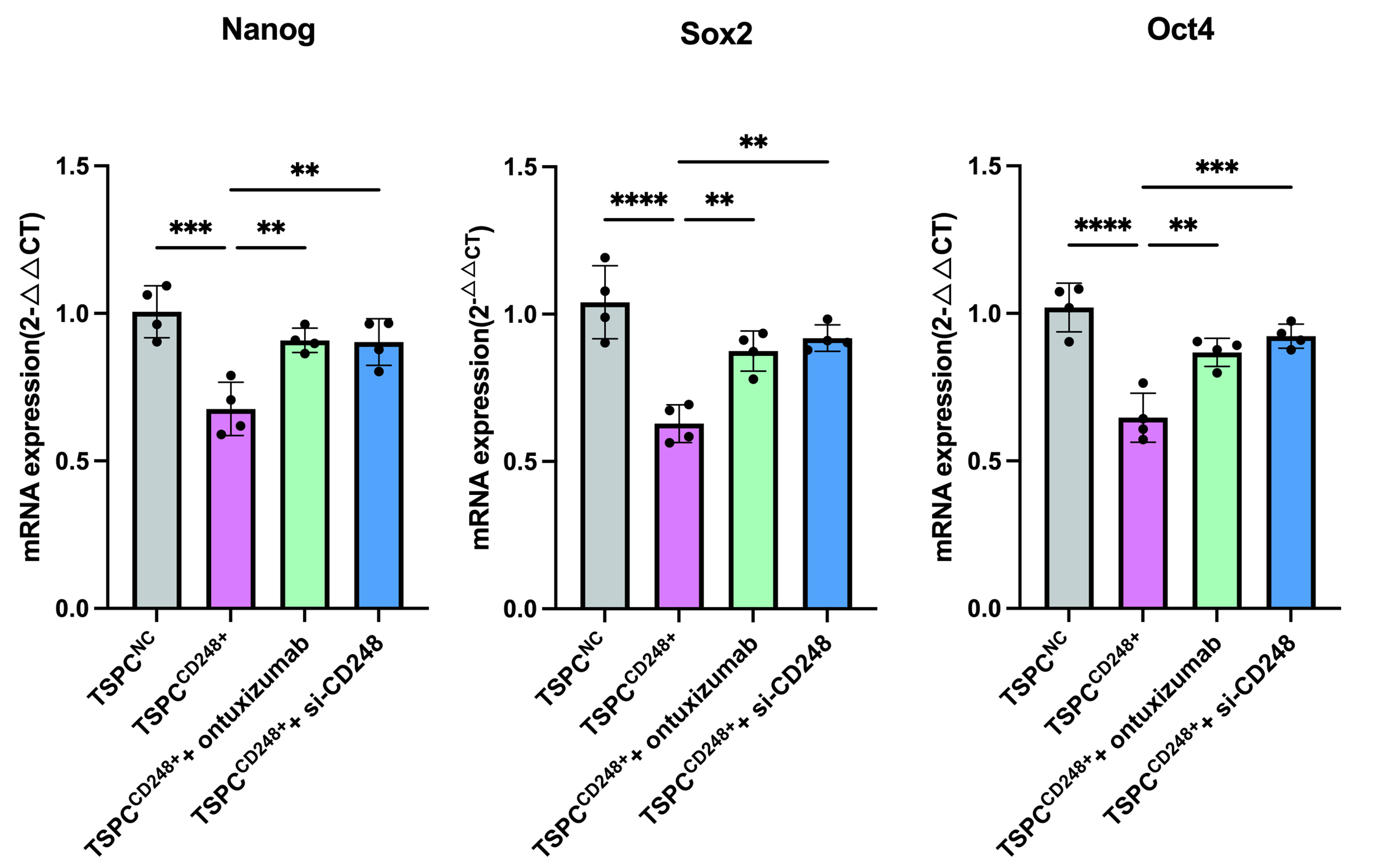


**Supplementary Figure 7. Validation of stem markers of TSPCs after different treatment**

Quantification of the qPCR results. The data are presented as the mean ± SD. *P < 0.05, **P < 0.01, ***P < 0.001, ****P < 0.0001.


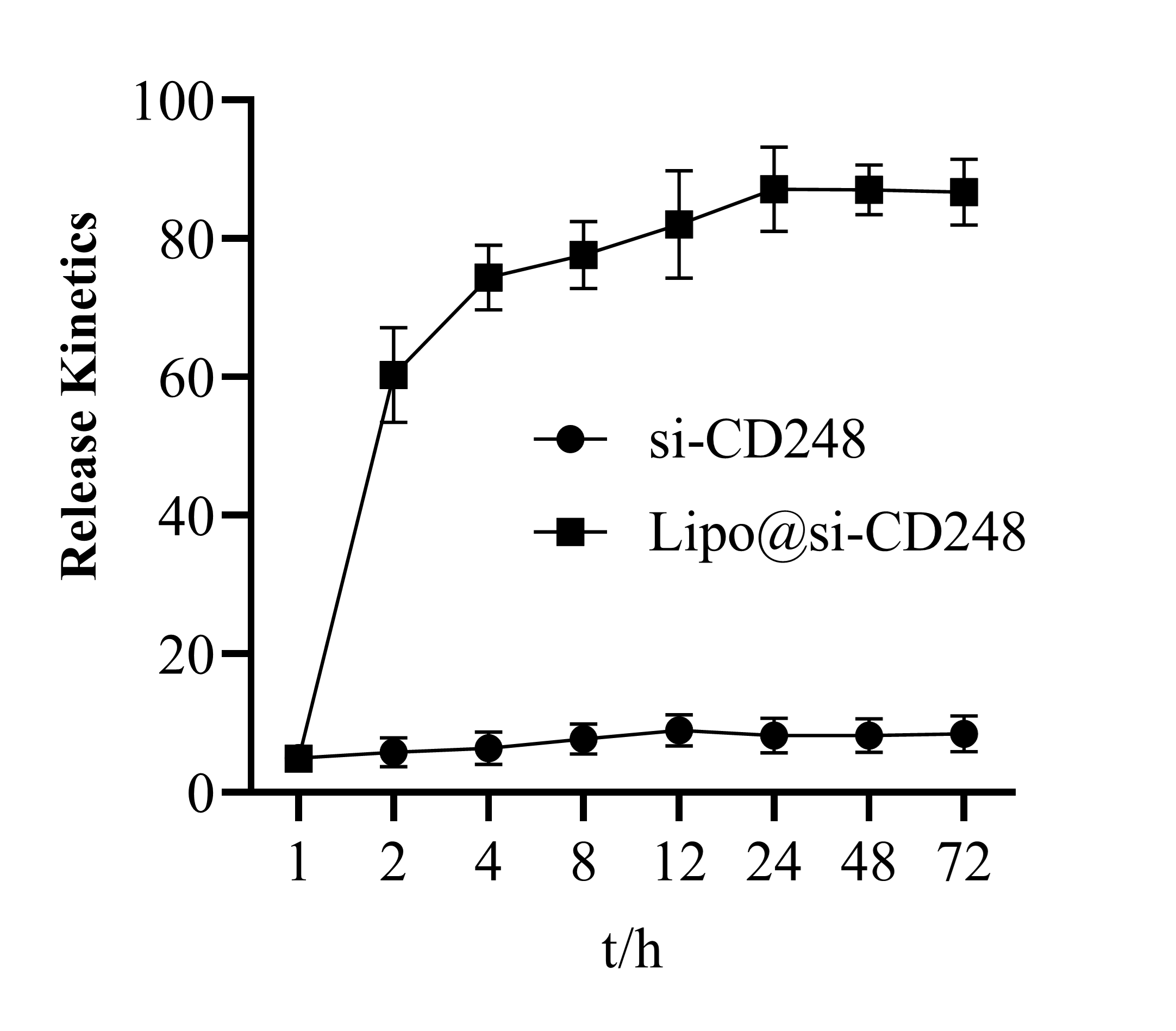


**Supplementary Figure 8. Degradation rate of si-RNA and lipo@si-CD248**


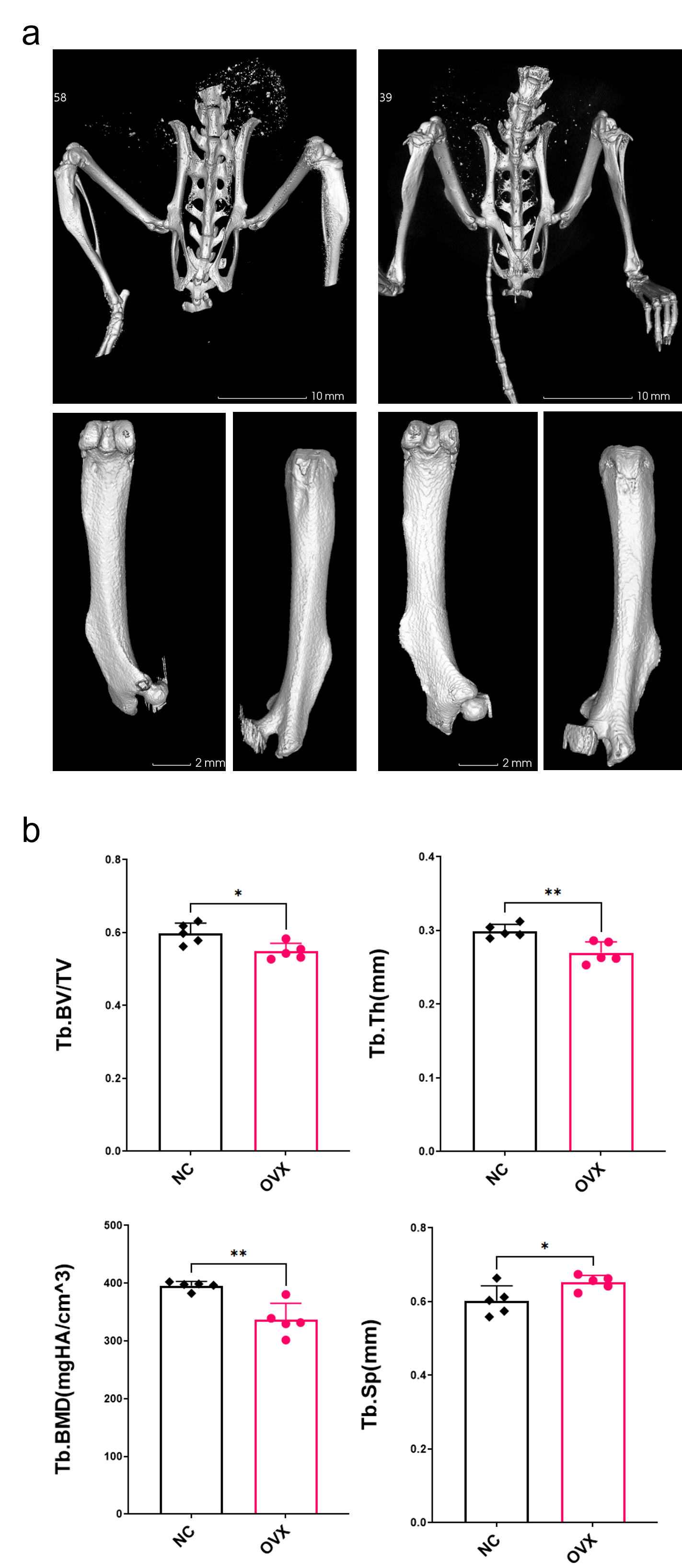


**Supplementary Figure 9. Micro-CT analysis of osteoporotic mice after 3 months of modeling**

**a.** Micro-CT images of the skeletal structure (upper row) and femurs (lower row) of normal control (NC) and ovariectomized (OVX) mice after 3 months of osteoporotic modeling. The images show a noticeable reduction in bone density and structural integrity in the OVX group compared to the NC group.

**b. Tb.BV/TV:** Trabecular bone volume fraction, showing a significant decrease in the OVX group compared to the NC group. **Tb.Th:** Trabecular thickness, which was significantly reduced in the OVX group. **Tb.BMD:** The trabecular bone mineral density was significantly lower in the OVX group. **Tb.Sp:** Trabecular separation, significantly increased in the OVX group. n=5, Data are presented as the mean ± SD. Significance was calculated with Student’s t test. *P < 0.05, **P < 0.01.
