# Supplementary material for: Bioactive siRNA‐Based Liposomes Promoted Tendon‐Bone Healing in Osteoporotic Mice by Recovering the Stemness of CD248+ TSPCs
Source: Adv Sci (Weinh). 2025 Jun 19;12(34):e09883. doi: 10.1002/advs.202509883 (PMC12442593; doi:10.1002/advs.202509883)
Supplement: Supplementary file 2 — Supplemental Table 1 [file ADVS-12-e09883-s002.docx]

| **Name** | **Forward primer 5’-3’** | **Reverse primer5’-3’** |
| --- | --- | --- |
| *GAPDH* | CGCTTCGCTCTCTGCTCCTCCTGT | GGTGACCAGGCGCCCAATACGA |
| *SOX2* | ACACCAA TCCCA TCCACACT | GCAAACTTCCTGCAAAGCTC |
| *NANOG* | TTCCTTCCTCCATGGATCTG | ATCTGCTGGAGGCTGAGGTA |
| *OCT4* | GAAGGATGTGGTCCGAGTGT | GTGAAGTGAGGGCTCCCATA |
